# Aging effects on DNA methylation modules in human brain and blood tissue

**DOI:** 10.1186/gb-2012-13-10-r97

**Published:** 2012-10-03

**Authors:** Steve Horvath, Yafeng Zhang, Peter Langfelder, René S Kahn, Marco PM Boks, Kristel van Eijk, Leonard H van den Berg, Roel A Ophoff

**Affiliations:** 1 Department of Human Genetics, David Geffen School of Medicine, University of California Los Angeles, Los Angeles, CA 90095, USA; 2 Department of Biostatistics, School of Public Health, University of California Los Angeles, Los Angeles, CA 90095, USA; 3 Department of Psychiatry, Rudolf Magnus Institute of Neuroscience, University Medical Center Utrecht, Utrecht, The Netherlands; 4 Department of Medical Genetics, University Medical Center Utrecht, Utrecht, The Netherlands; 5 Department of Neurology, University Medical Center Utrecht, Utrecht, The Netherlands; 6 UCLA Center for Neurobehavioral Genetics, Semel Institute of Neuroscience and Human Behavioral, School of Medicine, University of California Los Angeles, Los Angeles, CA 90095, USA

## Abstract

**Background:**

Several recent studies reported aging effects on DNA methylation levels of individual CpG dinucleotides. But it is not yet known whether aging-related consensus modules, in the form of clusters of correlated CpG markers, can be found that are present in multiple human tissues. Such a module could facilitate the understanding of aging effects on multiple tissues.

**Results:**

We therefore employed weighted correlation network analysis of 2,442 Illumina DNA methylation arrays from brain and blood tissues, which enabled the identification of an age-related co-methylation module. Module preservation analysis confirmed that this module can also be found in diverse independent data sets. Biological evaluation showed that module membership is associated with Polycomb group target occupancy counts, CpG island status and autosomal chromosome location. Functional enrichment analysis revealed that the aging-related consensus module comprises genes that are involved in nervous system development, neuron differentiation and neurogenesis, and that it contains promoter CpGs of genes known to be down-regulated in early Alzheimer's disease. A comparison with a standard, non-module based meta-analysis revealed that selecting CpGs based on module membership leads to significantly increased gene ontology enrichment, thus demonstrating that studying aging effects via consensus network analysis enhances the biological insights gained.

**Conclusions:**

Overall, our analysis revealed a robustly defined age-related co-methylation module that is present in multiple human tissues, including blood and brain. We conclude that blood is a promising surrogate for brain tissue when studying the effects of age on DNA methylation profiles.

## Background

Gene expression (messenger RNA transcript abundance) is modulated by epigenetic factors such as histone modifications, microRNAs, long noncoding RNAs, and DNA methylation. A large body of literature has provided evidence that age has a significant effect on cytosine-5 methylation within CpG dinucleotides [1-4]. A genome-wide decrease in DNA methylation has been shown to occur during *in vitro *aging [5] and *in vivo *aging [6,7]. Previous studies of aging effects on DNA methylation involved typically adults but recent studies also involved pediatric populations[8] Important insights have been gained regarding what types of genes show promoter hyper- or hypomethylation with age [9-11]. For example, early-life-induced programming that relies on DNA methylation appears to be at a considerable risk to become disrupted during aging [12,13]. Age-associated hypermethylation has been found to preferentially affect loci at CpG islands [14]. Important cancer related genes become hypermethylated during aging, including those encoding the estrogen receptor, insulin growth factor, and E-cadherin, and key developmental genes [9,15,16]. Rakyan *et al. *[15] showed that aging-associated DNA hypermethylation in blood occurs preferentially at bivalent chromatin domain promoters that are associated with key developmental genes. These genes are frequently hypermethylated in cancers, which points to a mechanistic link between aberrant hypermethylation in cancer and aging. Teschendorff *et al. *[16] identified a core DNA methylation signature of 589 CpGs that were significantly related to age. Further, the authors showed that Polycomb group protein targets (PCGTs) are far more likely to become methylated with age than non-targets (odds ratio = 5.3, *P *< 10^-10^), independently of sex, tissue type, disease state, and methylation platform. The authors identified a subset of 64 PCGTs exhibiting a clear trend toward hypermethylation with age across multiple cell types (blood, ovarian cancer, cervix, mesenchymal stem cells). This is a biologically important insight since gene repression by the PCG protein complex via histone H3 lysine 27 trimethylation (H3K27me3) is required for embryonic stem cell self-renewal and pluripotency [17,18]. While Teschendorff *et al. *evaluated the core aging signature in whole blood (WB), solid tissues, lung tissue, and cervix tissue, they did not include brain tissues.

In this study, we expand previous studies along multiple directions. First, we study aging effects in brain by evaluating aging effects in human tissue samples of the frontal cortex (FCTX), temporal cortex (TCTX), cerebellum (CRBLM), caudal pons (PONS) [19], prefrontal cortex [20], and mesenchymal stromal cells (Table 1). Second, we contrast aging effects on gene expression levels (mRNA) and DNA methylation levels and in brain and blood tissue. Third, we analyze four novel WB DNA methylation data sets involving *n *= 752 Dutch subjects. Fourth, we carry out a weighted correlation network analysis (WGCNA) of multiple methylation data sets. We apply the consensus module analysis to ten independent methylation data sets and identify a consensus co-methylation module (referred to as aging module) that contains CpG sites that are hypermethylated with age in multiple human tissues (WB, leukocytes, and different brain regions, including cortex, pons, and cerebellum). We then validate the presence of the aging co-methylation module in six additional independent data sets. Fifth, we demonstrate that the aging module found in adult populations can also be found in pediatric populations. Sixth, we demonstrate that an age association measure (based on membership to the aging module) leads to more pronounced biological insights than a standard meta-analysis measure that only considers marginal relationships between CpG sites and age.

**Table 1:** Description of DNA methylation data sets

Set	Analysis	n	Tissue	Description	Mean age	Age range	Platform	Reference	Public availability
1	Consensus	92	WB	Dutch controls from ALS study	64	34-88	Infin 27 k	Novel data	GSE41037
2	Consensus	273	WB	Dutch controls from SZ study	33	16-65	Infin 27 k	Novel data	GSE41037
3	Consensus	293	WB	Dutch cases, SZ	34	17-86	Infin 27 k	Novel data	GSE41037
4	Consensus	190	WB	Type 1 diabetics	44	24-74	Infin 27 k	[15]	GSE20067
5	Consensus	87	WB	Healthy older women	63	49-74	Infin 27 k	[14]	GSE20236
6	Consensus	261	WB	Healthy postmenopausal women from UKOPS	65	52-78	Infin 27 k	[15,33]	GSE19711
7	Consensus	132	FCTX	FCTX brain	48	16-101	Infin 27 k	[18]	GSE15745
8	Consensus	126	TCTX	TCTX brain	48	15-101	Infin 27 k	[18]	GSE15745
9	Consensus	123	PONS	PONS brain	46	15-101	Infin 27 k	[18]	GSE15745
10	Consensus	111	CRBLM	CRBLM brain	47	16-96	Infin 27 k	[18]	GSE15745
11	Validation	94	WB 450 k	Controls and SZ	32	18-65	Illumina 450 k	Novel data	GSE41169
12	Validation	24	MSCs	MSCs	50	21-85	Infin 27 k	[34,35]	GSE26519+GSE17448
13	Validation	50	CD14+CD4+	CD4+ T-cells and CD14+ monocytes	36	16-69	Infin 27 k	[14]	GSE20242
14	Validation	398	Leukocyte	Pediatric population	10	3-17	Infin 27 k	[24]	GSE27097
15	Validation	72	Leukocyte	Healthy children	5	1-16	Illumina 450 k	[24]	GSE36064
16	Validation	108	Prefrontal cortex	Healthy controls	26	-0.5-84	Infin 27 k	[19]	BrainCloudMethyl

Data sets 1 to 10 were used in the consensus network analysis while data sets 11 to 16 were used in the module validation (preservation) analysis. Our novel WB DNA methylation data sets (numbered 1 to 3 and 11) are composed of (*n *= 92 + 273 + 293 + 94) individuals. The study involved multiple tissues (blood, brain) and different populations (adults and healthy children). Note that the mean age (and age ranges) differ greatly across the studies. ALS, amyotrophic lateral sclerosis; CRBLM, cerebellum; FCTX, frontal cortex; MSC, mesenchymal stromal cell; PONS, caudal pons; SZ, schizophrenia; TCTX, temporal cortex; UKOPS, United Kingdom Ovarian Cancer Population Study; WB, whole blood.

## Results and discussion

### Advantages of DNA methylation over gene expression studies when it comes to studying aging effects across tissues

Given the difficulty of procuring human brain tissue versus the relative ease of measuring blood expression levels, a question of great practical importance is to determine to what extent blood tissue is a reasonable surrogate for brain tissue.

**Figure 1:**
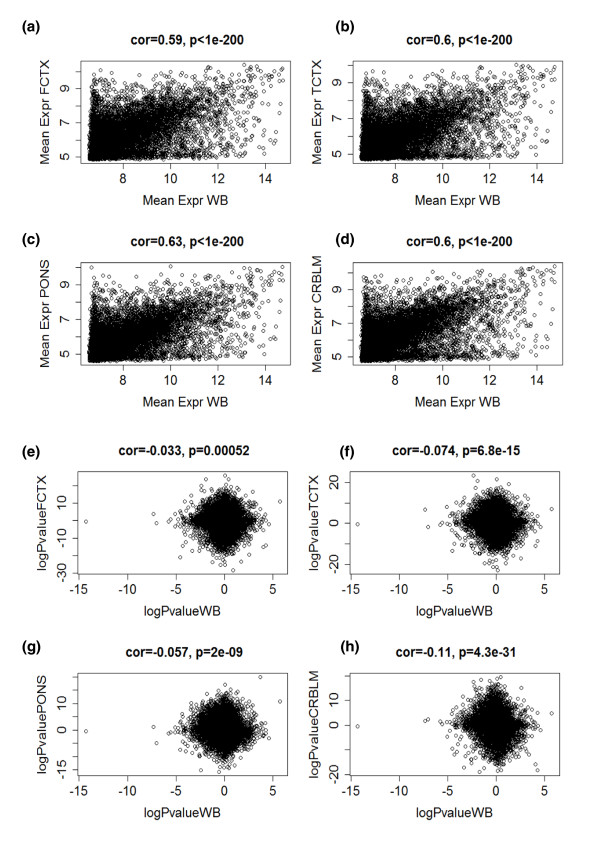
**Age effects on gene expression (mRNA) levels are not preserved between blood and brain tissue**. **(a-d) **Scatterplots of mean gene expression (mRNA abundance) in whole blood of the Dutch samples (x-axis) and corresponding mean brain expression values (y-axis) for frontal cortex (FCTX) (a), temporal cortex (TCTX) (b), pons (c), and cerebellum (CRBLM) (d). Each dot corresponds to a gene. The brain mRNA data (like the brain methylation data used in this article) were obtained from [19]. Note that only moderate correlations (around r = 0.6) exist between the mean expression values of these distinct tissues. **(e-g) **Overall age correlations of gene expression levels (mRNA) are not preserved between blood (x-axis) and brain tissues (y axes) as evidenced by the weak negative correlations reported in the title of each panel. The mRNA levels of each gene (represented by a dot) were correlated with subject age and a linear regression model was used to calculate a correlation test *P*-value. The x-axis of each scatterplot shows the (signed) logarithm (base 10) of the correlation test *P*-value in blood. Genes with a significant positive (negative) correlation with age have a high positive (negative) log *P*-value. The y-axis shows the corresponding correlation test *P*-values in the frontal cortex (e), temporal cortex (f), pons (g), and cerebellum (h).

**Figure 2:**
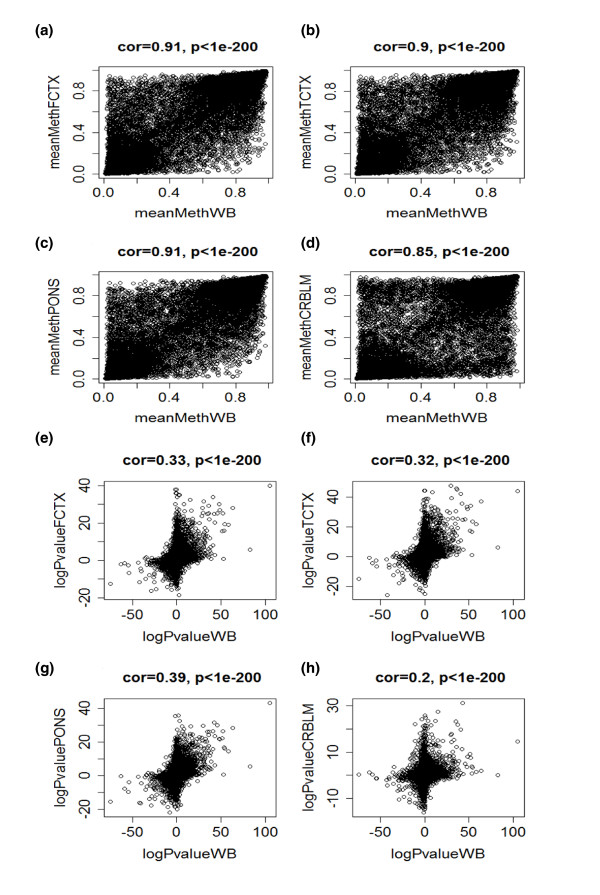
**Age effects on DNA methylation levels are well preserved between blood and brain tissue**. **(a-d) **Scatterplots of mean CpG methylation levels in whole blood of the Dutch samples (x-axis) and corresponding mean brain methylation values (y-axis) for frontal cortex (FCTX) (a), temporal cortex (TCTX) (b), pons (c), and cerebellum (CRBLM) (d). The brain methylation data used were obtained from [19]. Note that strong correlations (around r = 0.9) exist between the mean methylation levels in whole blood and brain tissue. We hypothesize that the relatively low correlation of r = 0.85 for cerebellum may reflect DNA quality. **(e-g) **Age correlations of CpG methylation levels show moderate preservation (correlations around 0.33) between blood (x-axis) and brain tissues (y axes). Analogous to Figure 1, the methylation levels of each gene (represented by a dot) were correlated with subject age and a linear regression model was used to calculate a correlation test *P*-value. The x-axis of each scatterplot shows the (signed) logarithm (base 10) of the correlation test *P*-value in blood. Genes with a significant positive (negative) correlation with age have a high positive (negative) log *P*-value. The y-axis shows the corresponding correlation test *P*-values in the frontal cortex (e), temporal cortex (f), pons (g), and cerebellum (h).

For gene expression studies (mRNA) the relationships are relatively weak. We and others have found that both mean gene expression levels and co-expression relationships are only weakly preserved between three brain regions and blood [21]. This is also demonstrated in Figure 1a-d, which presents scatterplots of mean gene expression (mRNA abundance) in WB versus corresponding mean brain expression values (y-axis) for frontal cortex, temporal cortex, pons, and cerebellum, respectively. Age effects on gene expression (mRNA) levels are not preserved between blood and brain tissue (Figures 1e-g). Given these negative results for mRNA, it is perhaps surprising that the results are much more encouraging for CpG methylation levels. Figure 2 shows that both mean methylation levels and age correlation test *P*-values are well preserved between blood and brain tissue. Figure 2a-d show that strong correlations (around r = 0.9) exist between the mean methylation levels in WB and brain tissues. Figure 2e-g show that age correlations of CpG methylation levels exhibit moderate preservation (correlations around 0.33) between blood and brain tissues.

Figures 1 and 2 suggest that gene expression (mRNA) levels are much more fluctuating compared to CpG methylation and therefore may be more 'noisy'. DNA methylation levels may be less variable and a better reflection of longer term environmental and genetic influences. These results led us to the hypothesis that one can identify co-methylation modules (clusters of CpG sites) that consistently relate to age across multiple tissues (consensus modules). To address this hypothesis, we collected both novel and published data as described in the following.

### DNA methylation data sets and clinical data description

Although many platforms exist for measuring methylation levels [22], the 16 DNA methylation data sets considered here were measured on the Illumina platform (Table 1). Data sets 1 through 10 were used in a consensus network analysis while the remaining data sets were used for validation. We analyzed 4 novel blood data sets (labeled 1 to 3, and 11) and 12 additional public data sets. While most of the first ten data sets used in the consensus network analysis involved healthy subjects, data set 3 involved blood tissue from schizophrenic cases. The effect of disease status on aging effects is discussed below and in Additional file 1. A more detailed description of the subject characteristics is provided in the Materials and methods section.

### Correlating CpG sites with age and standard meta analysis

Each individual CpG marker on the array was correlated to age in each of the ten data sets. We used a robust correlation measure (the biweight mid-correlation) and the Stouffer meta analysis approach (Materials and methods) to calculate a meta analysis *P*-value for each of the following data selections: i) the six WB data sets; ii) the four brain data sets; and iii) the ten data sets combined. Each *P*-value was log transformed (base 10) and multiplied by minus the sign of the correlation coefficients. For example, logPvalueWB takes on a large positive (negative) number for CpG probes that have a significant positive (negative) correlation with age across the six WB data sets. Analogously, logPvalueBrain and logPvalueAll measure age associations in the brain data sets and in all ten data sets, respectively.

Additional file 1 shows a scatterplot involving correlation test *P*-values for age effects in schizophrenia cases and healthy controls based on the Dutch WB data sets (data sets 2 and 3). Note that meta analysis *P*-values for schizophrenics (cases) are highly correlated (r = 0.78) with those of healthy controls (y-axis). Thus, Additional file 1 shows that schizophrenia disease status has a negligible effect on aging-related changes for the vast majority of CpG sites.

Additional file 2 shows scatterplots of correlation test *P*-values for measuring aging effects on DNA methylation profiles in the different brain regions (DNA methylation data sets 7 to 10). Overall, these *P*-values are highly correlated, which shows that age has a similar effect in all four brain regions. Having said this, comparisons involving the cerebellum (labeled CRBLM) show weaker correlations. Future studies involving additional cerebellum samples could address whether these systematic aging differences reflect the histologically distinct composition of the cerebellum or rather reflect sample quality issues such as degradation of DNA.

### Consensus module analysis with WGCNA

**Figure 3:**
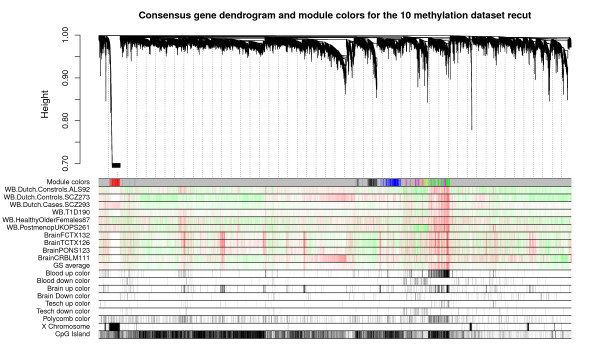
**Hierarchical cluster tree and consensus module structure**. Hierarchical cluster tree (dendrogram) of the consensus network based on ten independent methylation data sets. The first color band underneath the tree indicates the module color of each CpG site. The color grey is reserved for 'background' CpG sites that are not clustered into any module. The remaining color bands represent each gene's correlation with age in the underlying data sets; high intensity red values represent a strong positive correlation whereas high intensity green values represent a strong negative correlation. The remaining color bands indicate whether a gene was part of the core aging signature from Teschendorff *et al. *[16]. The color bands 'Tesch up' and 'Tesch down' indicate that Teschendorff *et al. *determined that methylation levels of this CpG site correlated positively or negatively with age, respectively. Other color bands indicate whether the CpG site is close to a known polycomb group target, is located on the X chromosome, or located in a CpG island. The figure suggests that the green module is composed of CpG sites that positively correlate with age in all ten tissues, which is why we refer to it as an aging module. Further, this aging related module is enriched with CpG sites that are close to Polycomb group target genes. Also note the presence of a very distinct red module that corresponds to CpG sites located on the X chromosome.

**Figure 4:**
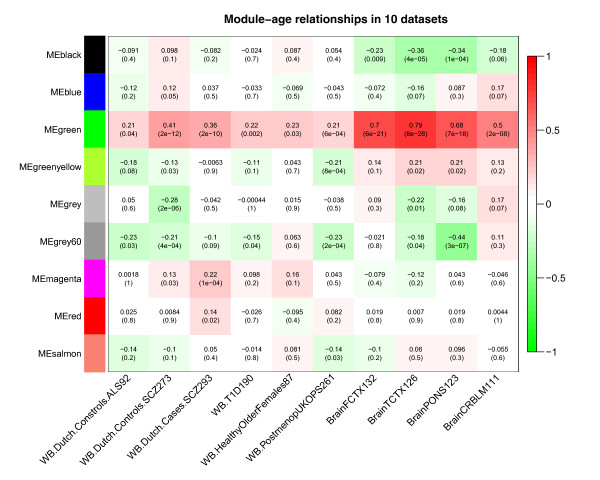
**Correlating consensus modules with age in the ten reference data sets**. Each row corresponds to a consensus co-methylation module (defined in Figure 3). More precisely, each row corresponds to the first principal component of each module (referred to as eigengene). The columns correspond to the age variable in each of the ten reference data sets. Each cell reports the correlation coefficient between the eigengene and age (top) and the corresponding *P*-value (bottom). Cells in the table are color coded using correlation values according to color scale on the right - that is, strong positive correlations are denoted by strong red color, and strong negative correlations by strong green color.

We used WGCNA to construct consensus modules across ten data sets (sets 1 to 10 in Table 1). Consensus modules group together methylation probes that are highly co-methylated across the ten input data sets (Materials and methods). Since consensus modules are, by definition, present in multiple independent data sets, they represent common (perhaps universal) and robust co-methylation relationships that reflect the underlying biology rather than technical artifacts. Weighted network methods are particularly useful for identifying consensus modules since they allow one to calibrate the individual networks. Further, they give rise to powerful module preservation statistics that can be used to determine whether modules can be validated in independent data sets [23,24]. Figure 3 shows the hierarchical cluster tree that results from consensus network analysis of sets 1 to 10. Branches in the tree correspond to consensus modules. The first color band underneath the tree indicates the module color of each CpG site. Note that the very distinct red module corresponds to CpG sites located on the X chromosome. Further, note that the green module is composed of CpG sites that positively correlate with age in all ten tissues, which is why we refer to it as the aging module. Figure 3 also indicates that this aging module is enriched with CpG sites that are close to PCGTs. The green module exhibits significant positive association with age in the ten reference data sets, as measured by the correlation of its eigengene with age in each of the ten data sets (Figure 4). The correlation is particularly high (r = 0.7) in the four brain data sets, which is due, in part, to the wide age range of the brain samples (Table 1).

### Validating the existence of the aging module in independent data sets

To assess the preservation of consensus modules (found in the reference data sets 1 to 10) in the additional validation data (data sets 11 to 16), we used the network module preservation statistics described in [24] and implemented in the R function modulePreservation in the WGCNA R package. Unlike traditional cross-tabulation statistics that rely on module matching between reference and test data sets, network preservation statistics do not require that modules be identified in the test data set, which has the major advantage that module preservation analysis is independent of the ambiguities associated with module identification in the test data set.

Results of the module preservation analysis in the validation data sets are reported in Additional file 3. Each figure (page) corresponds to a validation data set. The results show that the aging (green) module is highly preserved in the Illumina 450 K WB data set, which indicates that the module is not an artifact of the Illumina 27 K array. The aging module is also highly preserved in the blood cell type data (data set 13), the leukocyte data from pediatric subjects (data set 14), and healthy children (data set 15), and the prefrontal cortex (data set 16) but it is not preserved in the mesenchymal stromal cell (MSC) data set (data set 12). The lack of preservation in MSC data may be due to one of the following reasons. First, this was the smallest data set (*n *= 24). Second, it could reflect the fact that the human bone marrow MSCs were isolated from different locations (bone marrow aspirates or from the caput femoris upon hip fracture of elderly donors). Third, the MSC samples represent different cell passages from long-term culture. Thus, it is possible that the aging module will be observed in a larger MSC data set involving MSCs from a single location and a single cell passage.

**Figure 5:**
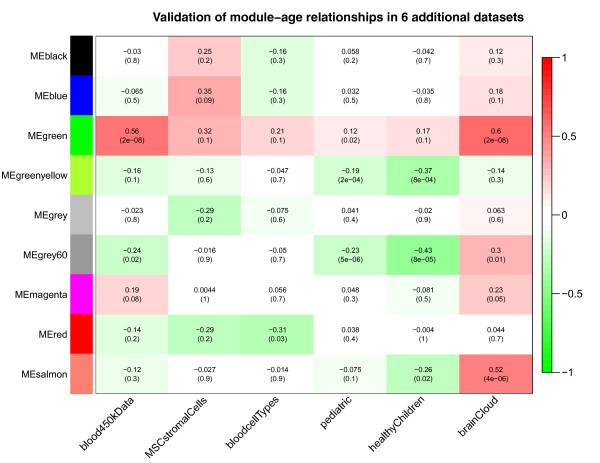
**Correlating consensus modules with age in the six validation data sets**. Each row corresponds to a consensus co-methylation module eigengene (defined in Figure 3). The columns correspond to the age variable in each of the six validation data sets. Each cell reports correlation coefficient between the eigengene and age (top) and the corresponding *P*-value (bottom). Cells in the table are color coded using correlation values according to color scale on the right. All of the reported modules were significantly preserved in the Dutch WB data measured on the Illumina 450 K array (Additional file 3). The green module has a particularly strong positive correlation with age in the Dutch 450 K blood data (r = 0.56, *P *= 2E-8) and in the brain cloud (pre-frontal cortex) data sets (r = 0.6, *P *= 2E-8). The age correlations for the green module are positive in all of the data sets (most of the marginally significant *P*-values reflect the low sample size in the respective data sets or the low age range).

Figure 5 reports the age correlations of all consensus modules in six validation data sets (data sets 11 to 16 in Table 1). The aging (green) module has a particularly strong positive correlation with age in the Dutch 450 K blood data (r = 0.56, *P *= 2E-8) and in the brain cloud (pre-frontal cortex) data sets (r = 0.6, *P *= 2E-8). The age correlations for the green module are positive in all of the data sets (most of the marginally significant *P*-values reflect the low sample size in the respective data sets or the narrow age range). Note that a one-sided correlation test *P*-value would be more appropriate in this validation step since the alternative hypothesis is that the correlation is less than zero. To arrive at one-sided *P*-values, divide the reported two-sided *P*-value by 2.

### Determinants of module membership in the (green) aging module

A major advantage of WGCNA is that it provides quantitative measures of module membership (referred to as module eigengene based connectivity, or kME; Materials and methods). Since kME.green(i) is defined as correlation between the i-th methylation probe and the green module eigengene, it takes on values between -1 and 1. The closer kME.green is to 1, the stronger the evidence that the probe is closely related to the green module. A CpG probe with kME.green = -1 has methylation levels that are perfectly anti-correlated with the module eigengene, that is, its methylation level is low when those of the module CpGs are highly methylated (and vice versa). Since the CpGs in the aging (green) module are positively correlated, CpGs with negative kME.green values are not part of the module. Here we characterize CpG probes (or genes) with high membership in the green aging module as well as the top probes identified in the meta-analysis of probe association with age (that is, probes with highest logPvalueAll). Specifically, we used marginal analysis as well as analysis of variance to relate kME.green and logPvalueAll to the following variables describing gene or sequence properties.

**Figure 6:**
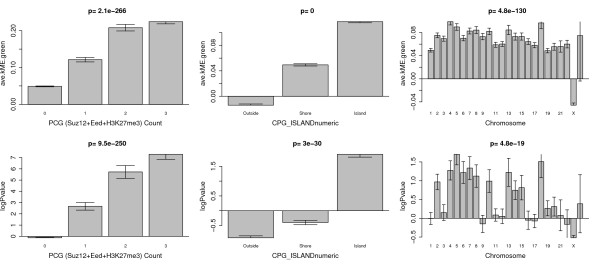
**Relating age relationships to chromosomal properties**. The bar plots in the top row relate average module membership in the aging module (average kME with respect to the green module) to Polycomb group (PCG) occupancy count, CpG island status, and chromosomal location, respectively. The bottom row shows the corresponding bar plots involving the (signed) logarithm of the meta analysis *P*-value. A positive (negative) log *P*-value indicates a positive (negative) age correlation of the CpG site. Both age association measures lead to the following results. First, the higher the PCG occupancy count, the stronger the age association. Second, CpG sites in CpG islands tend to have positive age correlations while those outside tend to have negative age correlations. Third, CpG sites on X chromosomes tend to have lower age correlations than those on other chromosomes. While both age association measures lead to similar conclusions, the results are more pronounced for the module membership measure (average kME), which suggests that this measure leads to more meaningful biological conclusions. Error bars indicate one standard error.

First, we studied occupancy counts for PCGTs since these targets are already known to have an increased chance of becoming methylated with age compared to non-targets [16]. Toward this end, we used the occupancy counts of Suz12, Eed, and H3K27me3 published in [18]. To obtain the protein binding site occupancy throughout the entire nonrepeat portion of the human genome, Lee *et al. *[17] isolated DNA sequences bound to a particular protein of interest (for example, Polycomb-group protein SUZ12) by immunoprecipitating that protein (chromatin immunoprecipitation) and subsequently hybridizing the resulting fragments to a DNA microarray. Figure 6 shows that the higher PCG occupancy count, the higher the average kME.green (Kruskal Wallis test *P *= 2.1 × 10^-266^) and the higher is the logPvalueAll (*P *= 9.5 × 10^-250^).

Second, we studied CpG island status (which is a discrete variable with three possible values: island, shore, outside of islands) since it has previously been reported that methylation probes with positive age correlation tend to be located inside CpG islands [14]. Figure 6 confirms this result: both average kME.green and logPvalueAll are significantly higher for probes located in CpG islands. For completeness, we note that PCG occupancy count and CpG island status (coded as a numeric variable) are weakly but significantly correlated (Spearman correlation = 0.14).

Third, we studied chromosomal location. Figure 6 reveals that the average kME.green is significantly lower (*P *= 4.8 × 10^-130^) for probes located on the X chromosome, that is, probes inside the aging module tend to be located on the autosomes. A similar (but less significant) tendency can be observed for logPvalueAll: probes with positive age correlations tend to be located on autosomes. We point out that this X chromosomal effect on module membership and the logPvalueAll could only be observed in data sets that contain both men and women (for example, the Dutch blood data and the brain data). A similar X chromosomal effect was observed in pediatric patients [8].

To explain this X chromosomal effect, we propose the following explanation: in data sets composed of both sexes, most X chromosomal probes have a much higher variance than autosomal probes due to the effect of X inactivation. Analysis of variance reveals that gender has typically a much stronger effect on the methylation levels of X-chromosomal probes than does age: across the 1,085 X chromosomal probes on the Illumina 27 K array, gender explains, on average, 57% of the variation while age explains only 0.9%. This dominant effect of gender on the methylation level of X chromosomal probes is also reflected by the presence of a very distinct X chromosomal module in data sets composed of both genders (Figure 3).

The above results demonstrate highly significant relationships between module membership and epigenetic variables. In the following, we probe deeper and determine the proportion of variance in module membership that can be explained by the epigenetic variables. Using analysis of variance (ANOVA), we can determine what proportion of the variation in eigengene-based connectivity kME can be explained by the different variables. As detailed in Table 2, the variables explain only 15.8% of the variation in kME.green. The two most significant variables (*P *< 2.2E-16) are Polycomb group (Suz12 + Eed + H3K27me3) occupancy count (which explains 7.1% of the variation) and CpG island status (7.3% of the variation). The proportion of variance explained (15.8%) is high considering that the ANOVA considered all 27 k probes on the Illumina 27 K platform while only 478 CpGs were part of the green consensus module. As a reference point, Table 2 also reports the results of ANOVA for explaining the variation in the signed logPvalueAll statistic (Stouffer's meta-analysis statistic described in our marginal analysis). In this case, the variables explain only 6.7% of the variation, which is substantially less than the 15.8% observed for module membership. These findings illustrate yet again that the module-based analysis in our study amplifies the biological signal inherent in the data.

**Table 2:** Analysis of variance of variance

Source of variation		ave.kME.green, total proportion of variance explained = 15.8%				logPvalue Stouffer, total proportion of variance explained = 6.7%		
			
Source	Degrees of freedom	Sums of Sq	Proportion of total variance	F statistic	*P*-value (F-test)	Sums of Sq	Proportion of total variance	*P*-value (F-test)
PCG (Suz12+Eed+H3K27me3) OccupancyCount	1	49.35	0.071	2013.0	< 2.2E-16	82,530	0.050	< 2.2E-16
CPG_Island	2	50.78	0.073	1035.7	< 2.2E-16	24,867	0.015	< 2.2E-16
X chromosome	1	9.74	0.014	397.4	< 2.2E-16	764	0.000	5.5E-04
Distance to TSS	1	3.50E-01	0.001	14.1	1.7E-04	1,827	0.001	9.3E-08
Residual error	23,903	586.02	0.842			1,529,855	0.933	
Total variation		696.24				1,639,843		

The first six columns detail the sources of variation on ave.kME with respect to the green (aging) module. Polycomb group occupancy count explains the highest proportion of the total variance followed by CpG island status (inside, outside, and shore). Comparing the results from ANOVA applied to average kME with those from applying it to the log*P*-value (significance measure resulting from Stouffer's meta-analysis) reveals marked differences in the proportion of variance explained (15.8% versus 6.7%), which suggests again that the average kME measure is biologically more meaningful than the meta-analysis *P*-value. TSS, transcription start site.

### Functional enrichment of aging module genes based on gene ontolgy categories

To understand the biological meaning of the aging (green) module, we carried out several functional enrichment analyses of the 1,000 CpG sites with the highest average module membership value (kME) to the green module. These top 1,000 CpG sites are reported in Additional file 4. Recall that these CpG sites are typically located in promoters of corresponding genes whose gene symbols are also located in Additional file 4. Additional file 4 also allows the user to access information on the CpGs that make up the aging (green) module. Specifically, this Excel file reports a) the Illumina CpG probe identifier, b) the corresponding gene symbol, and c) importantly, the average module membership with respect to the green module. Thus, the reader can simply choose the top 100, 500 or 1,000 genes with highest module membership with respect to this aging module. We find that the measure of module membership is highly robust and largely unaffected by the branch cutting procedure used to define the green module.

The gene symbols corresponding to the top 1,000 most connected green module CpGs were used as input of the gene ontology (GO) enrichment analysis tool DAVID (but our results are highly robust with respect to the number of input genes).

Additional file 5 shows the results of a GO enrichment analysis using the DAVID software when 'GO Chart' output is selected. It shows that the most significant enrichment is achieved for the Swiss Protein Interaction Resource keyword 'developmental protein' (*P*-value 8.9E-37).

Notable enrichment categories include neuron differentiation (*P *= 8.5E-26), neuron development (*P *= 9.6E-17), and DNA-binding (*P *= 2.3E-21).

Additional file 6 shows the results of a GO enrichment analysis using the DAVID software when 'GO Cluster' output is selected. Notable enrichment categories include DNA-binding region: Homeobox (*P *= 7.6E-29), neuron differentiation (*P *= 8.5E-26), neuron development (*P *= 9.6E-17), cell fate commitment (*P *= 2.8E-19), embryonic morphogenesis (*P *= 2.4E-15), and regulation of transcription from RNA polymerase II promoter (*P *= 1.4E-11).

As a caveat, we mention that none of these GO categories are specific to aging.

### Enrichment analysis with respect to cell markers

To study the properties of lists of genes whose promoters contain CpG sites that are part of the aging (green) module, we also used the userListEnrichment function [25] (which is part of the WGCNA R package) since it contains lists of known marker genes for blood, brain, and stem cell types. This function was used to assess whether the top 1,000 module genes in the aging module (that is, genes with highest average kMEgreen) are significantly enriched (hypergeometric test) in brain-, blood- and stem cell-related lists curated from the literature.

As detailed in Additional file 7, the most significant *P*-value (hypergeometric *P*-value 2.5E-113) was achieved for lists of genes identified to play a role for polycomb in human embryonic stem cells [18]. Highly significant enrichment was found for genes bound by Suz12 (*P *= 5.3E-106; genes reported in Table S8 of [18]) and genes known to be occupied by Suz12, Eed and H3K27me (*P *= 2.5E-113; genes reported in Table S9 from Lee *et al. *[17]).

Since the consensus modules were identified in brain tissues (in addition to blood) it comes as no surprise that the gene list was also enriched (*P *= 9.8E-09) for neuronal cell type markers reported by Cahoy [26]. Age-related DNA methylation changes in the human cerebral cortex are known to involve differentiated neurons [27]. But future follow-up studies involving additional data are clearly warranted to explore why neuronal-specific enrichments can be found.

Strikingly, the analysis revealed significant (Bonferroni corrected *P *= 0.0016) enrichment for genes (*CDH13*, *GDF10*, *NTRK3*, *PENK*, *RBP1*, *RBP4*, *UCHL1*, *WIF1*) whose expression values are known to be downregulated in early Alzheimer's disease [28]. Age is one of the biggest risk factors for developing Alzheimer's disease [29]. While DNA methylation is known to play a role in age-related neurodegenerative diseases such as Alzheimer's disease [30,31], our results indicate that a methylation footprint may also be found in blood tissue.

## Conclusions

A summary and overview of our consensus network analysis is presented in Additional file 8. The consensus network analysis based on WGCNA reveals the existence of a robustly defined co-methylation module that consistently relates to age in human brain and blood tissue. Our analysis indicates that this consensus module-based analysis leads to biologically more meaningful results. GO enrichment analysis reveals that the module is composed of CpG sites in promoters of key development genes and genes that are known to play a role in cellular aging in brain and blood tissue. The good preservation over tissues, consistent association with age and meaningful biology shows that blood is a promising surrogate for brain tissue when studying the effects on age on DNA methylation profiles.

## Materials and methods

### DNA methylation data description

Table 1 describes the 16 human DNA methylation data sets that were used in this study. The table reports the sample size, mean age, age range, and tissue source (mostly blood and brain tissue), citation and, where available, the GSE identifier of the data set in the Gene Expression Omnibus (GEO) database.

Our four novel data sets (labeled 1 to 3 and 11) measure methylation levels in WB containing polymorphonuclear leukocytes, mononuclear cells, platelets and red blood cells. It is worth mentioning that platelets and red blood cells do not contain nuclear DNA.

Samples were collected between 1 January 2004 and 31 December 2007 at the University Medical Center Utrecht, a referral clinic in the Netherlands. Specifically, data set 1 was composed of 92 healthy Dutch subjects who had been collected as healthy controls for a case control study of amyotrophic lateral sclerosis. To be clear, these were healthy control samples, that is, amyotrophic lateral sclerosis patients were not included in our study. Data sets 2 and 3 corresponded to 273 healthy controls and 293 diseased individuals, respectively, from a case-control study of schizophrenia. We found aging correlations were highly preserved (0.78) between schizophrenia cases and controls (Additional file 2). By separating the cases from controls into distinct and separate data sets in our meta analysis, we conditioned on disease status. Our novel validation data set number 11 was measured on the Illumina Infinium HumanMethylation450 BeadChip array and contained WB measurements from both healthy control subjects and schizophrenia samples (roughly half cases, half controls). The new data presented in this article are available from the GEO repository GSE41037. Also, they can be downloaded from our webpage [32].

Data sets 4 to 10 and 12 to 16 were downloaded from the GEO repository (see the GEO accession numbers in Table 1).

Data set 4 (type 1 diabetes) consists of WB samples from 190 individuals (93 women and 97 men) with a mean age of 44 years (range 24 to 74 years) [16]. Since all samples were type 1 diabetics (no controls), we were not able to evaluate whether type 1 diabetes status has an effect on aging relationships. Since it is likely that disease status adds additional heterogeneity to studies of aging effects on methylation levels, including this data set is expected to bias the analysis towards the null hypothesis. But we find that the reported age relationships can also be observed in this data set, that is, the data set contains a relevant biological signal.

Data set 5 (healthy older women) consists of 87 WB samples from women whose mean age was 63 years (range 49 to 74) [15]. The samples were collected from different healthy females (both twin pairs and singletons).

Data set 6 (healthy postmenopausal women) consists of 261 WB samples from women with a mean of 65 years (range 52 to 78) [16,33]. While the data come from the United Kingdom Ovarian Cancer Population Study (UKOPS), it is important to emphasize we include only the healthy controls in our study.

Data sets 7 to 10 (different brain regions) consist of tissue samples of the frontal cortex (FCTX), temporal cortex (TCTX), cerebellum (CRBLM) and caudal pons (PONS) obtained from 150 subjects whose mean age was 49 years (range 15 to 101) [19]. These individuals, who had donated their brains for research, were of non-Hispanic, Caucasian ethnicity, and none had a clinical history of neurological or cerebrovascular disease, or a diagnosis of cognitive impairment during life. Demographics, tissue source and cause of death for each subject are reported in [19]. Removal of potential outliers (as described in the following section on sample pre-processing) reduced the number of retained samples to between 111 and 132 (Table 1).

Data sets 1 to 10 were used to construct the consensus networks while data sets 11 to 16 were used to validate the existence of the module.

Data set 11 (WB on the Illumina 450 K array from controls and schizophrenic cases) is described above. By using a different Illumina version we were able to establish that our aging module can also be detected using a different (newer) array.

Data set 12 (MSCs; also known as mesenchymal stem cells) comprise a subpopulation of multipotent adult stem cells that is able to differentiate into diverse mesodermal cell lineages. MSCs are concurrently tested in a large number of clinical trials for a wide range of therapeutic applications surrounding regenerative medicine. The MSCs from human bone marrow were either isolated from bone marrow aspirates or from the caput femoris upon hip fracture of elderly donors [34]. The MSCs from adipose tissue were isolated from lipoaspirates [35]. In our analysis, we ignored the fact that the MSCs come from different tissues (bone marrow, adipose) and that they represent different cell passages from long-term culture. While Schellenberg *et al. *[35] showed that MSCs from different tissues exhibit considerable differences in their DNA methylation profiles, their growth pattern, immunophenotype and *in vitro *differentiation potential are quite similar. Hence, we ignored this sample heterogeneity since our consensus analysis had shown that the aging module could be detected in diverse tissues (blood and brain). We expect that the module would show even stronger age correlations and preservation if the analysis was restricted to MSCs from a single tissue origin. Further, cell passage status (related to cellular senescence status) was ignored in our validation analysis since senescence status did not affect the majority of CpG cites: only 517 senescence-associated CpG sites were identified in [35].

Data set 13 (CD14+ monocytes and CD4+ T cells) consists of sorted CD4+ T cells and CD14+ monocytes from blood of an independent cohort of 25 healthy subjects [15]. CD14+ monocytes derive from the myeloid lineage and can live several weeks. CD4+ T cells derive from the lymphoid lineage and represent a variety of cell types with longer life spans (from months to years).

Data set 14 (leukocytes from a pediatric population) consists of peripheral blood leukocyte samples from 398 healthy males from the Simons Simplex Collection, who are siblings of autism spectrum disorder individuals but do not present a clinical diagnosis of this disorder [8]. To be clear, these individuals can be considered healthy controls. At the time of collection, these individuals had an age range from 3 to 17 years (mean 10 years).

Data set 15 (leukocytes from healthy children) is another pediatric group from [8] (independent from data set 14 described above). This study involved 72 peripheral blood leukocyte samples from healthy males collected from Children's Hospital Boston [8].

Data set 16 (prefrontal cortex from healthy controls) consists of 108 samples (mean age 26 years ranging from samples before birth up to age 84 years) [20]. These post-mortem human brains from non-psychiatric controls were collected at the Clinical Brain Disorders Branch (National Institute of Mental Health). The DNA methylation data are publicly available from the webpage of the standalone package BrainCloudMethyl.

### DNA methylation profiling and pre-processing steps

Full experimental methods and detailed descriptions of these public data sets can be found in the original references. Here we briefly summarize the main steps. Methylation analysis was performed either using the Illumina Infinium Human Methylation27 BeadChip [36] or the Illumina Infinium HumanMethylation450 BeadChip. The Illumina HumanMethylation27 BeadChips measures bisulfite-conversion-based, single-CpG resolution DNA methylation levels at 27,578 different CpG sites within 5' promoter regions of 14,475 well-annotated genes in the human genome. Data from the two platforms were merged by focusing on the roughly 26 k CpG sites that are present on both platforms. We followed the standard protocol of Illumina methylation assays, which quantifies methylation levels by the β value using the ratio of intensities between methylated (signal A) and unmethylated (signal B) alleles. Specifically, the β value was calculated from the intensity of the methylated (M corresponding to signal A) and unmethylated (U corresponding to signal B) alleles, as the ratio of fluorescent signals β = Max(M,0)/[Max(M,0) +Max(U,0) + 100]. Thus, β values range from 0 (completely unmethylated) to 1 (completely methylated) [37].

As an unbiased, high level outlier detection approach we use the inter-array correlation and formed a measure of sample network connectivity (based on the sum of interarray correlations). Samples whose inter-array connectivity was significantly lower (*P *< 0.01) than the average observed inter-array connectivity were removed from the data set. Specifically, outlier detection and removal was performed using an iterative process of removing outliers with average inter-array correlation ≤ 2 standard deviations below the mean until visual inspection of the cluster dendrogram and plot of the mean inter-array correlation revealed no further outliers.

### Dealing with polymorphic and non-specific CpGs

Some CpG probes are known to contain common SNPs, which can affect the measure of methylation level [38]. To evaluate whether the green aging module contains such polymorphic CpGs (that is, CpGs that are overlapping SNPs), we used an updated table from Chen *et al. *[38] composed of 875 CpGs that were found by downloading the entire dbSNP build 132 and then mapping it against the Illumina 27 probes based on chromosomal position. Fortunately, it turns out that our aging module is significantly (*P *= 0.00020) under-enriched for these polymorphic CpGs. Only 11 of the 1,000 most connected green module CpGs are known to contain a SNP as indicated in Additional file 4. The under-enrichment makes sense since polymorphic CpGs are unlikely to show a strong age relationship due to the affects of the genetic variation.

We also evaluated whether CpGs in the aging module are non-specific (that is, whether their sequences map to highly homologous genomic sequences) since between 6% and 10% of probes on the Illumina 27 K array are non-specific [38]. We found no significant relationship between membership to the aging module and non-specificity (defined using a table from [38]). Additional file 4 also indicates which of the green module CpGs are non-specific.

### Dealing with batch effects

Batch effects are known to influence DNA methylation levels. In our study, batches can arise due to Illumina plate effects or due to the independent data sets generated by different labs. To protect against spurious artifacts due to batch effects, we used the following approaches. First, our network analysis used a consensus module approach which implicitly conditions on each data set by aggregating the information of ten individual networks (one for each of the ten data sets). Modules due to plate effects (or other batch effects) in one data set cannot be found in other data sets, that is, they will not give rise to consensus modules. By definition, consensus modules can be observed in the majority of the ten data sets, that is, they are highly reproducible across multiple data sets (generated by different labs). Second, we only considered those consensus modules that could also be found in data generated by the Illumina 450 K array (which we generated in one batch). Thus, the reported modules are highly reproducible in the Illumina 27 K and 450 K arrays. Third, we validate the presence and age correlations of our green aging module in multiple independent data sets. A module reflecting a spurious batch effect or other technical artifact would not validate in independent validation data sets.

### Statistical analysis

#### Meta analysis relating methylation probes to age

We used the *metaAnalysis *R function from the WGCNA library to carry out a meta-analysis of aging effects across multiple data sets. Given methylation (or other) data from multiple independent data sets, and the corresponding ages, the function was used to calculate Stouffer's meta-analysis Z statistics (reviewed in [39]), *P*-values, and corresponding q-values (local false discovery rates) [40]. Briefly, Stouffer's approach for combining multiple correlation test statistics across the data sets is based on calculating the following meta-analysis Z statistic:

$$metaZ=\frac{\overset{no.dataSets}{\underset{s=1}{\sum }}w_{s}Z_{s}}{\sqrt{\overset{no.dataSets}{\underset{s=1}{\sum }}{\left(w_{s}\right)}^{2}}}$$

where w_s _denotes a weight associated with the s-th data set.

We found the results were similar irrespective of the weights, which is why we focused on the equal weight method (w_i = 1).

#### Consensus network analysis with WGCNA

An R software tutorial that describes these methods can be found at the following webpage [32].

Co-expression methodology is typically used for studying relationships between gene expression levels [41]. Here we use these techniques for studying relationships between methylation levels. To describe the relationships among methylation profiles, we used WGCNA. Compared to unweighted network methods, WGCNA has the following advantages: first, it preserves the continuous nature of co-methylation information [42,43]; second, weighted networks are particularly useful for consensus module detection since they allow one to calibrate the individual networks; third, they give rise to powerful module preservation statistics (described below).

The consensus network analysis was applied to data sets 1 to 10 described in Table 1. For each data set, a signed weighted network adjacency matrix is defined as:

$$a_{ij}={\left|\frac{1+cor\left(x_{i},x_{j}\right)}{2}\right|}^{b}$$

where *x_i _*is the methylation profile of the i-th CpG site (probe on the array), that is, *x_i _*is a numeric vector whose entries report the β values across the individuals. Note that the adjacency *a_ij _*is a number between 0 and 1 that is a monotonically increasing function of the correlation coefficient. The power *b *is a soft-thresholding parameter that can be used to emphasize high positive correlations at the expense of low correlations. We chose the default threshold of 12. A major advantage of weighted correlation networks is that they are highly robust with regard to the choice of *b *[42]. While WGCNA can be applied to one data set at a time to identify co-methylation modules, we aimed to define shared 'consensus' modules that are present in the first ten data sets described in Table 1. To address this challenge, we used a consensus network approach that identifies modules that are present in all data sets. The goal of our consensus network analysis was i) to identify modules shared across the ten independent data sets, and ii) calculate representatives of each module (module eigengenes), iii) correlate module eigengenes with age, and iv) define a continuous measure of module membership in the consensus module (referred to as kME).

Briefly, each of the ten network adjacency matrices were transformed into the topological overlap matrix (TOM). TOM is a highly robust measure of interconnectedness and is widely used for clustering network nodes into modules [44,45]. The element TOM_ij _of the topological overlap matrix measures the direct connection between the network nodes (in this case, methylation profiles) i and j as well as the extent to which they share common network neighbors. Studies of our group [42,45-48] and of independent groups [49] provide empirical evidence that the topological overlap measure is a biologically meaningful network similarity measure. To ensure comparability of the ten TOMs, we calibrated them by raising the entries of each matrix to fixed power. The power was chosen so that each TOM had the same 95% percentile (roughly the same maximum value). Note that this calibration step only works for a weighted network, which highlights the utility of weighted networks for the purpose of consensus network analysis.

The consensus topological overlap measure (CTOM) was defined as the lower quartile across the ten calibrated topological overlap measures, CTOM_ij _= quartile(TOM_ij_^(1)^, TOM_ij_^(2)^, ..., TOM_ij_^(10)^), where TOM_ij_^(s) ^is the calibrated topological overlap of nodes i and j in set s, s = 1, 2, ... 10. The lower quartile is a conservative choice, for example, a CTOM value of 0.5 indicates that in 75% of the data sets the TOM connection strength between the two CpGs is ≥ 0.5. Our robustness study with respect to different quantile choices (from minimum to median) shows that the green aging module can be found irrespective of the quantile choice. The reader can explore the effect of different consensus module approaches using our posted R software tutorials.

Average linkage hierarchical clustering was applied to the consensus dissimilarity measure DissCTOM defined as DissCTOMij = 1 - CTOMij. Modules (branches of the resulting clustering tree) were subsequently identified using the adaptive branch cutting approach implemented in R package dynamicTreeCut [50].

Since each module groups together highly correlated methylation profiles, it is useful to summarize the profiles in each module using a single representative profile. Here we use the module eigengene [47], defined as the first principal component of the module methylation matrix. For each module, its module eigengene can be used to define a measure of module membership, denoted kME, which quantifies how close a methylation profile is to the module. Specifically, for each methylation profile and each module, kME is defined as the correlation of the methylation profile with the module eigengene. Defining module membership as correlation allows one to easily calculate the statistical significance (*P*-value) of each module membership. In turn, this makes it possible to use standard meta-analysis techniques (for example, the aforementioned Stouffer method) to aggregate the module memberships across the ten data sets. Here, we used the average aggregation implemented in the WGCNA function consensusKME. Module membership measures allow one to efficiently annotate all methylation profiles on the array [51].

Further details on the consensus module approach can be found in [23,47].

Numerous network inference algorithms have been developed, including ARACNE [52] and BANJO [53]. A comparison of different network inference algorithms lies beyond the scope of this biology paper. A recent review article compares the performance of WGCNA to ARACNE and other algorithms [49]. Advantages of WGCNA include i) that it provides module preservation statistics that are being used in this article, ii) powerful functions for consensus module analysis, iii) the availability of module membership measures, and iv) proven methods for finding modules.

#### Module preservation analysis

Our module preservation analysis is based on the approach described in [24] and implemented in the modulePreservation R function implemented in the WGCNA R package. The modulePreservation R function implements several powerful network-based statistics for evaluating module preservation.

For each module in the reference data (for example, a brain methylation data set) one observes a value of a module preservation statistic in the test data (for example, the MSC methylation data set). An advantage of these network-based preservation statistics is that they make few assumptions regarding module definition and module properties. Traditional cross-tabulation-based statistics are inferior for the purposes of our study. While cross-tabulation approaches are intuitive, they have several disadvantages. To begin with, they are only applicable if the module assignment in the test data results from applying a module detection procedure to the test data. Even when modules are defined using a module detection procedure, cross-tabulation-based approaches face potential pitfalls. A module found in the reference data set will be deemed non-reproducible in the test data set if no matching module can be identified by the module detection approach in the test data set. Such non-preservation may be called weak non-preservation: 'the module cannot be found using the current parameter settings of the module detection procedure'. On the other hand, here we are interested in establishing strong non-preservation: 'the module cannot be found irrespective of the parameter settings of the module detection procedure'. Strong non-preservation is difficult to establish using cross-tabulation approaches that rely on module assignment in the test data set. A second disadvantage of a cross-tabulation-based approach is that it requires that for each reference module one finds a matching test module. This may be difficult when a reference module overlaps with several test modules or when the overlaps are small. A third disadvantage is that cross-tabulating module membership between two networks may miss the fact that the patterns of density or connectivity between module nodes are highly preserved between the two networks. The correlation network-based statistics implemented in the modulePreservation function do not require the module assignment in the test network but require the user to input DNA methylation data underlying a reference data set and a test data set.

The specific nature of correlation networks allows us to use a permutation test for calculating four density preservation statistics (summarized by Zdensity), three connectivity-based statistics (summarized by Zconnectivity), and a composite summary preservation statistic Zsummary. In our application, Zdensity worked well at showing that the aging module was preserved while Zconnectivity (and therefore Zsummary) did reveal evidence of preservation. Thus, while the density (average adjacency) of the aging module is preserved, hub gene status is much less preserved.

Therefore, Additional file 3 presents the statistic Zdensity that quantifies whether the density patterns of modules defined in the ten reference data sets are preserved in a given test data set. We adopted the suggested significance thresholds described in [24]: Zdensity < 2 implies no evidence for module preservation, 2 < Zdensity < 10 implies weak to moderate evidence, and Zdensity > 10 implies strong evidence for module preservation. Thus, we report Zdensity for each consensus module in each of the six validation data sets. The module preservation statistics proposed in [24] are defined for a single reference and a single test data set. Since our consensus modules were identified in an analysis of ten data sets, for the purposes of module preservation calculation we have ten reference data sets. To arrive at a single preservation score for each test set, we averaged the module preservation statistics across the ten data sets. Thus, the calculation of the module preservation statistic followed the following steps. The module preservation function was applied to the k-th (k = 1, ..., 10) reference set and the given test (validation) set to calculate Zdensity(k). Next, the ten module preservation statistics Zdensity(k) values were averaged.

The permutation based Z statistics often depend on the module size (that is, the number of CpGs in a module). This fact reflects the intuition that it is more significant to observe that the connectivity patterns among hundreds of nodes are preserved than to observe the same among say only five nodes. Having said this, there will be many situations when the dependence on module size is not desirable, such as when preservation statistics of modules of different sizes are to be compared. In this case, it is useful to use the composite module preservation statistic medianRank for comparing relative preservation among multiple modules: a module with lower median rank tends to exhibit stronger observed preservation statistics than a module with a higher median rank. Since medianRank is based on the observed preservation statistics (as opposed to Z statistics or other permutation test statistics), we find that it is much less dependent on module size.

A major step involved in testing whether consensus modules that were defined with respect to the Illumina 27 K array (data sets 1 to 10) could also be detected using the 450 K array (validation data set 11). It turned out that roughly half of the modules did not show evidence of module preservation (see the first panel in Additional file 3).

The lack of module preservation for half of the modules is probably not due to sample size (since *n *= 92 is moderately large) or due to batch effects (since the 450 K data were generated in one batch). Instead, the following reasons may explain the relatively low preservation. First, it could reflect that half of the samples were schizophrenics. Since we find that schizophrenia status has only a very minor effect, we think this explanation is unlikely. Second, lack of preservation could reflect that there are systematic differences due to the different platforms and sample preparation steps. This is possible since unsupervised hierarchical clustering analysis based on interarray correlations reveals that samples measured on the 450 K platform are globally distinct from those on the 27 K platform even when only the roughly 26 K overlapping probes are being used.

To be safe, modules that did not show evidence of module preservation in the Illumina 450 K data were removed from the analysis. To avoid confusing the reader with two module assignments (before and after carrying out the module preservation analysis in data set 11), Figures 3 and 4 only depict those consensus modules that also showed significant evidence of preservation in data set 11. Thus, the resulting modules were identified using a very conservative approach: not only are these modules present in ten data sets involving different tissues but they also validated across Illumina platforms. Notably, the aging related module was highly preserved (Additional file 3).

#### WGCNA software

The freely available statistical analysis software (WGCNA R package) and R tutorials for constructing a weighted gene co-expression network are described in [54]. Consensus network analysis was carried out with R function '*blockwiseConsensusModules*' in the WGCNA R package [54].

Our online R software tutorial easily permits the user to identify tissue-specific age related modules and CpGs.

#### Gene ontology enrichment analysis

The functional enrichment of gene lists was evaluated in two ways. First, we used the on-line functional annotation tool DAVID [55]. DAVID functionally categorizes gene lists based on enrichment for GO, Kyoto Encyclopedia of Genes and Genomes (KEGG), SwissProt terms, and other biological knowledge databases. For each gene list, the software returns *P*-values for assessing significance of overlaps with known functional categories. We used DAVID to characterize genes corresponding to modules and to lists of genes that are positively (or negatively) related to age.

Second, we used the function userListEnrichment from the WGCNA library to find enrichment for cell type markers and other brain-related categories [25].

## Abbreviations

ANOVA: analysis of variance; CTOM: consensus topological overlap measure; GEO: Gene Expression Omnibus; GO: gene ontology; kME: connectivity based on the module eigengene, also known as module membership; MSC: mesenchymal stromal cell; PCGT: Polycomb group protein target; SNP: single nucleotide polymorphism; TOM: topological overlap matrix; WB: whole blood; WGCNA: weighted correlation network analysis.

## Competing interests

The authors declare that they have no competing interests.

## Authors' contributions

SH and RO conceived of the study. SH, MPB, and RO wrote the article. RO, RSK, and LHV contributed the four novel DNA methylation data sets. YZ, SH, and KVE analyzed the methylation data. PL and SH contributed R software code. All authors have read and approved the manuscript for publication.

## Supplementary Material

Additional file 1 **Schizophrenia status has a negligible effect on aging effects**. A scatterplot of correlation test *P*-values for correlations between age and methylation profiles in schizophrenia cases (x-axis) and healthy controls (y-axis) based on the Dutch whole blood data sets (data sets 2 and 3). Additional file 1 shows that schizophrenia disease status has a negligible effect on aging-related changes for the vast majority of CpG sites. Click here for file

Additional file 2 **Age effects in different brain regions**. Scatterplots of correlation test *P*-values for correlations between age and methylation profiles in the four brain regions (data sets 7 to 10). Overall, these *P*-values are highly correlated, which shows that age has a similar effect in all four brain regions. Click here for file

Additional file 3 **Module preservation analysis**. The figures report the results of the module preservation analysis in the validation data sets. Each figure (page) corresponds to one validation data set. The left and right panels of each figure show the results for the Zdensity and medianRank statistics, respectively. The higher the value of the Zdensity statistic (and the lower the value of the median rank statistic), the stronger the evidence that the consensus module (based on the ten reference data sets) is preserved in the validation data set. The Zdensity statistic is based on a permutation test that allows one to establish significance thresholds (that are indicated by the horizontal lines at values 2 and 5 in the left panel). Values of Zdensity larger than 5 indicate moderate preservation while values below 2 indicate no evidence of preservation. Click here for file

Additional file 4 **1,000 CpGs with highest average module membership in the green aging module**. The comma delimited file reports the Illumina array probe identifiers of the 1,000 CpG sites with highest average module membership (kME) with respect to the aging-related (green) module. This table also reports the average kME value and the gene symbols of neighboring genes. Further, it contains additional probe annotations. Column SNPpolymorphicCpGfromChen2011 indicates which of the CpGs is known to contain a common SNP [38]. Column NumberOfMatchingBasesToCrossReactiveTarget indicates which CpGs are non-specific (NA means it is specific) according to [38]. Click here for file

Additional file 5 **Gene ontology enrichment chart of the 1,000 aging module**. The Excel table shows the results of a gene ontology enrichment analysis using the DAVID software when 'GO Chart' output is selected. Click here for file

Additional file 6 **Gene ontology enrichment cluster of the 1,000 aging module**. The Excel table shows the results of a gene ontology enrichment analysis using the DAVID software when 'GO Cluster' output is selected. Click here for file

Additional file 7 **Enrichment analysis using the userListEnrichment function**. The comma delimited file shows the results of a gene list enrichment analysis using the userListEnrichment function [25]. This function was used to assess whether the top 1,000 aging-related module genes (highest average kMEgreen) are significantly enriched (hypergeometric test) with genes that are part of the brain-, blood- and stem cell-related lists curated from the literature. The userListEnrichment function was used to study the properties of lists of genes whose promoters contain CpG sites that are part of the aging related (green) module. Click here for file

Additional file 8 **Analysis overview**. The figure shows the analysis steps of the consensus network analysis and their rationale. Click here for file

## References

[B1] Guarente L Do changes in chromosomes cause aging? Cell 1996 13 9 12 10.1016/S0092-8674(00)80072-0 8689691

[B2] Wareham KA Lyon MF Glenister PH Williams ED Age related reactivation of an X-linked gene. Nature 1987 13 725 727 10.1038/327725a0 3600770

[B3] Berdyshev G Korotaev G Boiarskikh G Vaniushin B Nucleotide composition of DNA and RNA from somatic tissues of humpback and its changes during spawning. Biokhimiia 1967 13 88 993 5628601

[B4] Bell JT Tsai P-C Yang T-P Pidsley R Nisbet J Glass D Mangino M Zhai G Zhang F Valdes A Shin S-Y Dempster EL Murray RM Grundberg E Hedman AK Nica A Small KS Dermitzakis ET McCarthy MI Mill J Spector TD Deloukas P The Mu TC Epigenome-Wide Scans Identify Differentially Methylated Regions for Age and Age-Related Phenotypes in a Healthy Ageing Population. PLoS Genet 2012 13 e1002629 10.1371/journal.pgen.1002629 22532803 PMC3330116

[B5] Wilson V Jones P DNA methylation decreases in aging but not in immortal cells. Science 1983 13 1055 1057 10.1126/science.6844925 6844925

[B6] Bjornsson HT Sigurdsson MI Fallin MD Irizarry RA Aspelund T Cui H Yu W Rongione MA Ekström TJ Harris TB Launer LJ Eiriksdottir G Leppert MF Sapienza C Gudnason V Feinberg AP Intra-individual Change Over Time in DNA Methylation With Familial Clustering. JAMA: The Journal of the American Medical Association 2008 13 2877 2883 10.1001/jama.299.24.2877 18577732 PMC2581898

[B7] Boks MP Derks EM Weisenberger DJ Strengman E Janson E Sommer IE Kahn RS Ophoff RA The Relationship of DNA Methylation with Age, Gender and Genotype in Twins and Healthy Controls. PLoS ONE 2009 13 e6767 10.1371/journal.pone.0006767 19774229 PMC2747671

[B8] Alisch RS Barwick BG Chopra P Myrick LK Satten GA Conneely KN Warren ST Age-associated DNA methylation in pediatric populations. Genome Res 2012 13 623 632 10.1101/gr.125187.111 22300631 PMC3317145

[B9] Fraga MF Agrelo R Esteller M Cross-Talk between Aging and Cancer. Annals of the New York Academy of Sciences 2007 13 60 74 10.1196/annals.1395.005 17460165

[B10] Fraga MF Esteller M Epigenetics and aging: the targets and the marks. Trends in Genetics 2007 13 413 418 10.1016/j.tig.2007.05.008 17559965

[B11] Rodríguez-Rodero S Fernández-Morera J Fernandez A Menéndez-Torre E Fraga M Epigenetic regulation of aging. Discov Med 2010 13 225 233 20875344

[B12] Mugatroyd C Wu Y Bockmühl Y Spengler D The Janus face of DNA methylation in aging. AGING 2010 2 10.18632/aging.100124 PMC2850147 20354272

[B13] Murgatroyd C Patchev AV Wu Y Micale V Bockmuhl Y Fischer D Holsboer F Wotjak CT Almeida OFX Spengler D Dynamic DNA methylation programs persistent adverse effects of early-life stress. Nat Neurosci 2009 13 1559 1566 10.1038/nn.2436 19898468

[B14] Christensen B Houseman E Marsit C Zheng S Wrensch M Wiemels J Nelson H Karagas M Padbury J Bueno R Sugarbaker D Yeh R Wiencke J Kelsey K Aging and Environmental Exposures Alter Tissue-Specific DNA Methylation Dependent upon CpG Island Context. PLoS Genet 2009 13 e1000602 10.1371/journal.pgen.1000602 19680444 PMC2718614

[B15] Rakyan VK Down TA Maslau S Andrew T Yang TP Beyan H Whittaker P McCann OT Finer S Valdes AM Leslie RD Deloukas P Spector TD Human aging-associated DNA hypermethylation occurs preferentially at bivalent chromatin domains. Genome Res 2010 13 434 439 10.1101/gr.103101.109 20219945 PMC2847746

[B16] Teschendorff AE Menon U Gentry-Maharaj A Ramus SJ Weisenberger DJ Shen H Campan M Noushmehr H Bell CG Maxwell AP Savage DA Mueller-Holzner E Marth C Kocjan G Gayther SA Jones A Beck S Wagner W Laird PW Jacobs IJ Widschwendter M Age-dependent DNA methylation of genes that are suppressed in stem cells is a hallmark of cancer. Genome Res 2010 13 440 446 10.1101/gr.103606.109 20219944 PMC2847747

[B17] Boyer LA Plath K Zeitlinger J Brambrink T Medeiros LA Lee TI Levine SS Wernig M Tajonar A Ray MK Bell GW Otte AP Vidal M Gifford DK Young RA Jaenisch R Polycomb complexes repress developmental regulators in murine embryonic stem cells. Nature 2006 13 349 353 10.1038/nature04733 16625203

[B18] Lee TI Jenner RG Boyer LA Guenther MG Levine SS Kumar RM Chevalier B Johnstone SE Cole MF Isono K-i Koseki H Fuchikami T Abe K Murray HL Zucker JP Yuan B Bell GW Herbolsheimer E Hannett NM Sun K Odom DT Otte AP Volkert TL Bartel DP Melton DA Gifford DK Jaenisch R Young RA Control of Developmental Regulators by Polycomb in Human Embryonic Stem Cells. Cell 2006 13 301 313 10.1016/j.cell.2006.02.043 16630818 PMC3773330

[B19] Gibbs JR van der Brug MP Hernandez DG Traynor BJ Nalls MA Lai S-L Arepalli S Dillman A Rafferty IP Troncoso J Johnson R Zielke HR Ferrucci L Longo DL Cookson MR Singleton AB Abundant Quantitative Trait Loci Exist for DNA Methylation and Gene Expression in Human Brain. PLoS Genet 2010 13 e1000952 10.1371/journal.pgen.1000952 20485568 PMC2869317

[B20] Numata S Ye T Hyde Thomas M Guitart-Navarro X Tao R Wininger M Colantuoni C Weinberger Daniel R Kleinman Joel E Lipska Barbara K DNA Methylation Signatures in Development and Aging of the Human Prefrontal Cortex. The American Journal of Human Genetics 2012 13 260 272 10.1016/j.ajhg.2011.12.020 PMC3276664 22305529

[B21] Cai C Langfelder P Fuller TF Oldham MC Luo R van den Berg LH Ophoff RA Horvath S Is human blood a good surrogate for brain tissue in transcriptional studies? BMC Genomics 2010 13 589 10.1186/1471-2164-11-589 20961428 PMC3091510

[B22] Stolzenberg DS Grant PA Bekiranov S Epigenetic methodologies for behavioral scientists. Hormones and Behavior 2011 13 407 416 10.1016/j.yhbeh.2010.10.007 20955712 PMC3093106

[B23] Horvath S Weighted Network Analysis Applications in Genomics and Systems Biology 2011 Springer

[B24] Langfelder P Luo R Oldham MC Horvath S Is My Network Module Preserved and Reproducible? PLoS Comput Biol 2011 13 e1001057 10.1371/journal.pcbi.1001057 21283776 PMC3024255

[B25] Miller JA Cai C Langfelder P Geschwind DH Kurian SM Salomon DR Horvath S Strategies for aggregating gene expression data: The collapseRows R function. BMC Bioinformatics 2011 13 322 10.1186/1471-2105-12-322 21816037 PMC3166942

[B26] Cahoy JD Emery B Kaushal A Foo LC Zamanian JL Christopherson KS Xing Y Lubischer JL Krieg PA Krupenko SA Thompson WJ Barres BA A Transcriptome Database for Astrocytes, Neurons, and Oligodendrocytes: A New Resource for Understanding Brain Development and Function. The Journal of Neuroscience 2008 13 264 278 10.1523/JNEUROSCI.4178-07.2008 18171944 PMC6671143

[B27] Siegmund KD Connor CM Campan M Long TI Weisenberger DJ Biniszkiewicz D Jaenisch R Laird PW Akbarian S DNA Methylation in the Human Cerebral Cortex Is Dynamically Regulated throughout the Life Span and Involves Differentiated Neurons. PLoS ONE 2007 13 e895 10.1371/journal.pone.0000895 17878930 PMC1964879

[B28] Parachikova A Agadjanyan MG Cribbs DH Blurton-Jones M Perreau V Rogers J Beach TG Cotman CW Inflammatory changes parallel the early stages of Alzheimer disease. Neurobiology of aging 2007 13 1821 1833 10.1016/j.neurobiolaging.2006.08.014 17052803 PMC2198930

[B29] Swerdlow RH Is aging part of Alzheimer's disease, or is Alzheimer's disease part of aging? Neurobiology of aging 2007 13 1465 1480 10.1016/j.neurobiolaging.2006.06.021 16876913

[B30] Groen T Tollefsbol TO DNA Methylation and Alzheimer's Disease Epigenetics of Aging. 2010 Springer New York 315 326

[B31] Irier H Jin P Dynamics of DNA Methylation in Aging and Alzheimer's Disease. DNA Cell Biol 2012 10.1089/dna.2011.1565 PMC3461976 22313030

[B32] Aging related methylation modules: R software tutorials and data. http://www.genetics.ucla.edu/labs/horvath/CoexpressionNetwork/Methylation/AgeModule

[B33] Song H Ramus SJ Tyrer J Bolton KL Gentry-Maharaj A Wozniak E Anton-Culver H Chang-Claude J Cramer DW DiCioccio R Dork T Goode EL Goodman MT Schildkraut JM Sellers T Baglietto L Beckmann MW Beesley J Blaakaer J Carney ME Chanock S Chen Z Cunningham JM Dicks E Doherty JA Durst M Ekici AB Fenstermacher D Fridley BL Giles G A genome-wide association study identifies a new ovarian cancer susceptibility locus on 9p22.2. Nat Genet 2009 13 996 1000 10.1038/ng.424 19648919 PMC2844110

[B34] Bork S Pfister S Witt H Horn P Korn B Ho A Wagner W DNA methylation pattern changes upon long-term culture and aging of human mesenchymal stromal cells. Aging Cell 2010 13 54 63 10.1111/j.1474-9726.2009.00535.x 19895632 PMC2814091

[B35] Schellenberg A Lin Q Schuler H Koch C Joussen S Denecke B Walenda G Pallua N Suschek C Zenke M Wagner W Replicative senescence of mesenchymal stem cells causes DNA-methylation changes which correlate with repressive histone marks. Aging (Albany NY) 2011 13 873 888 22025769 10.18632/aging.100391 PMC3227452

[B36] Weisenberger D den Berg D Pan F Berman B Laird P Comprehensive DNA methylation analysis on the Illumina Infinium assay platform. Technical report Illumina, Inc, San Diego 2008 http://www.illumina.com/Documents/products/appnotes/appnote_infinium_methylation.pdf

[B37] Dunning M Barbosa-Morais N Lynch A Tavare S Ritchie M Statistical issues in the analysis of Illumina data. BMC Bioinformatics 2008 13 85 10.1186/1471-2105-9-85 18254947 PMC2291044

[B38] Chen Y Choufani S Ferreira J Grafodatskaya D Butcher D Weksberg R Sequence overlap between autosomal and sex-linked probes on the Illumina HumanMethylation27 microarray. Genomics 2011 13 214 222 10.1016/j.ygeno.2010.12.004 21211562

[B39] Whitlock M Combining probability from independent tests: the weighted Z-method is superior to Fisher's approach. J Evolutionary Biology 2005 13 1368 10.1111/j.1420-9101.2005.00917.x 16135132

[B40] Storey JD Tibshirani R Statistical significance for genomewide studies. Proceedings of the National Academy of Sciences of the United States of America 2003 13 9440 9445 10.1073/pnas.1530509100 12883005 PMC170937

[B41] Almaas E Biological impacts and context of network theory. J Exp Biol 2007 13 1548 1558 10.1242/jeb.003731 17449819

[B42] Zhang B Horvath S A general framework for weighted gene co-expression network analysis. Statistical Applications in Genetics and Molecular Biology 2005 4 10.2202/1544-6115.1128 16646834

[B43] Horvath S Zhang B Carlson M Lu KV Zhu S Felciano RM Laurance MF Zhao W Qi S Chen Z Lee Y Scheck AC Liau LM Wu H Geschwind DH Febbo PG Kornblum HI Cloughesy TF Nelson SF Mischel PS Analysis of oncogenic signaling networks in glioblastoma identifies ASPM as a molecular target. Proceedings of the National Academy of Sciences 2006 13 17402 17407 10.1073/pnas.0608396103 PMC1635024 17090670

[B44] Ravasz E Somera AL Mongru DA Oltvai ZN Barabasi AL Hierarchical organization of modularity in metabolic networks. Science 2002 13 1551 1555 10.1126/science.1073374 12202830

[B45] Yip AM Horvath S Gene network interconnectedness and the generalized topological overlap measure. BMC Bioinformatics 2007 13 22 10.1186/1471-2105-8-22 17250769 PMC1797055

[B46] Song L Langfelder P Horvath S Comparison of co-expression measures: mutual information, correlation, and model based indices. UCLA Technical Report Submitted 2012 10.1186/1471-2105-13-328 PMC3586947 23217028

[B47] Langfelder P Horvath S Eigengene networks for studying the relationships between co-expression modules. BMC Systems Biology 2007 13 54 10.1186/1752-0509-1-54 18031580 PMC2267703

[B48] Li A Horvath S Network neighborhood analysis with the multi-node topological overlap measure. Bioinformatics 2007 13 222 231 10.1093/bioinformatics/btl581 17110366

[B49] Allen J Xie Y Chen M Girard L Xiao G Comparing Statistical Methods for Constructing Large Scale Gene Networks. PLoS ONE 2012 13 e29348 10.1371/journal.pone.0029348 22272232 PMC3260142

[B50] Langfelder P Zhang B Horvath S Defining clusters from a hierarchical cluster tree: the Dynamic Tree Cut library for R. Bioinformatics 2007 November:btm563 10.1093/bioinformatics/btm563 18024473

[B51] Horvath S Dong J Geometric Interpretation of Gene Coexpression Network Analysis. PLoS Comput Biol 2008 13 e1000117 10.1371/journal.pcbi.1000117 18704157 PMC2446438

[B52] Margolin A Nemenman I Basso K Wiggins C Stolovitzky G Favera R Califano A ARACNE: An Algorithm for the Reconstruction of Gene Regulatory Networks in a Mammalian Cellular Context. BMC Bioinformatics 2006 13 S7 16723010 10.1186/1471-2105-7-S1-S7 PMC1810318

[B53] Smith V Yu J Smulders T Hartemink A Jarvis E Computational Inference of Neural Information Flow Networks. PLoS Computational Biology 2006 2 10.1371/journal.pcbi.0020161 PMC1664702 17121460

[B54] Langfelder P Horvath S WGCNA: an R package for weighted correlation network analysis. BMC Bioinformatics 2008 13 559 10.1186/1471-2105-9-559 19114008 PMC2631488

[B55] Hosack DA Dennis G Jr Sherman BT Lane HC Lempicki RA Identifying biological themes within lists of genes with EASE. Genome Biol 2003 13 R70 10.1186/gb-2003-4-10-r70 14519205 PMC328459

