# Supplementary figures and images for: Aging effects on DNA methylation modules in human brain and blood tissue
Source: Genome Biol. 2012 Oct 3;13(10):R97. doi: 10.1186/gb-2012-13-10-r97 (PMC4053733; doi:10.1186/gb-2012-13-10-r97)

**Blood:  $\log(\text{Pvalue}[\text{controls}])$  vs  $\log(\text{Pvalue}[\text{SCZ cases}])$ , cor: 0.779**

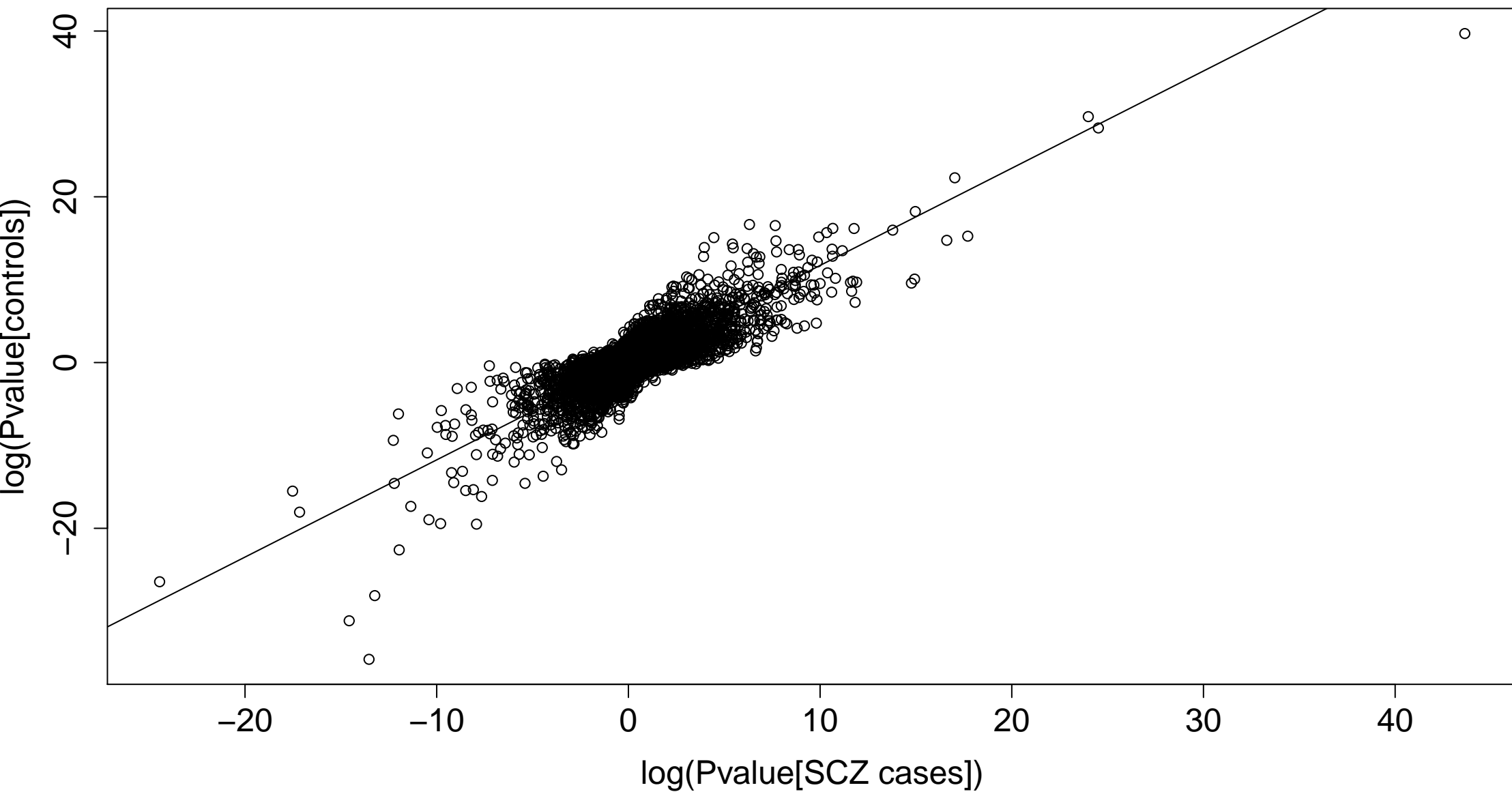

Supplement: Additional file 1 — Schizophrenia status has a negligible effect on aging effects. A scatterplot of correlation test P-values for correlations between age and methylation profiles in schizophrenia cases (x-axis) and healthy controls (y-axis) based on the Dutch whole blood data sets (data sets 2 and 3). Additional file 1 shows that schizophrenia disease status has a negligible effect on aging-related changes for the vast majority of CpG sites. [file gb-2012-13-10-r97-S1.PDF]
