# Supplementary material for: Aging effects on DNA methylation modules in human brain and blood tissue
Source: Genome Biol. 2012 Oct 3;13(10):R97. doi: 10.1186/gb-2012-13-10-r97 (PMC4053733; doi:10.1186/gb-2012-13-10-r97)
Supplement: Additional file 2 — Age effects in different brain regions. Scatterplots of correlation test P-values for correlations between age and methylation profiles in the four brain regions (data sets 7 to 10). Overall, these P-values are highly correlated, which shows that age has a similar effect in all four brain regions. [file gb-2012-13-10-r97-S2.PDF]

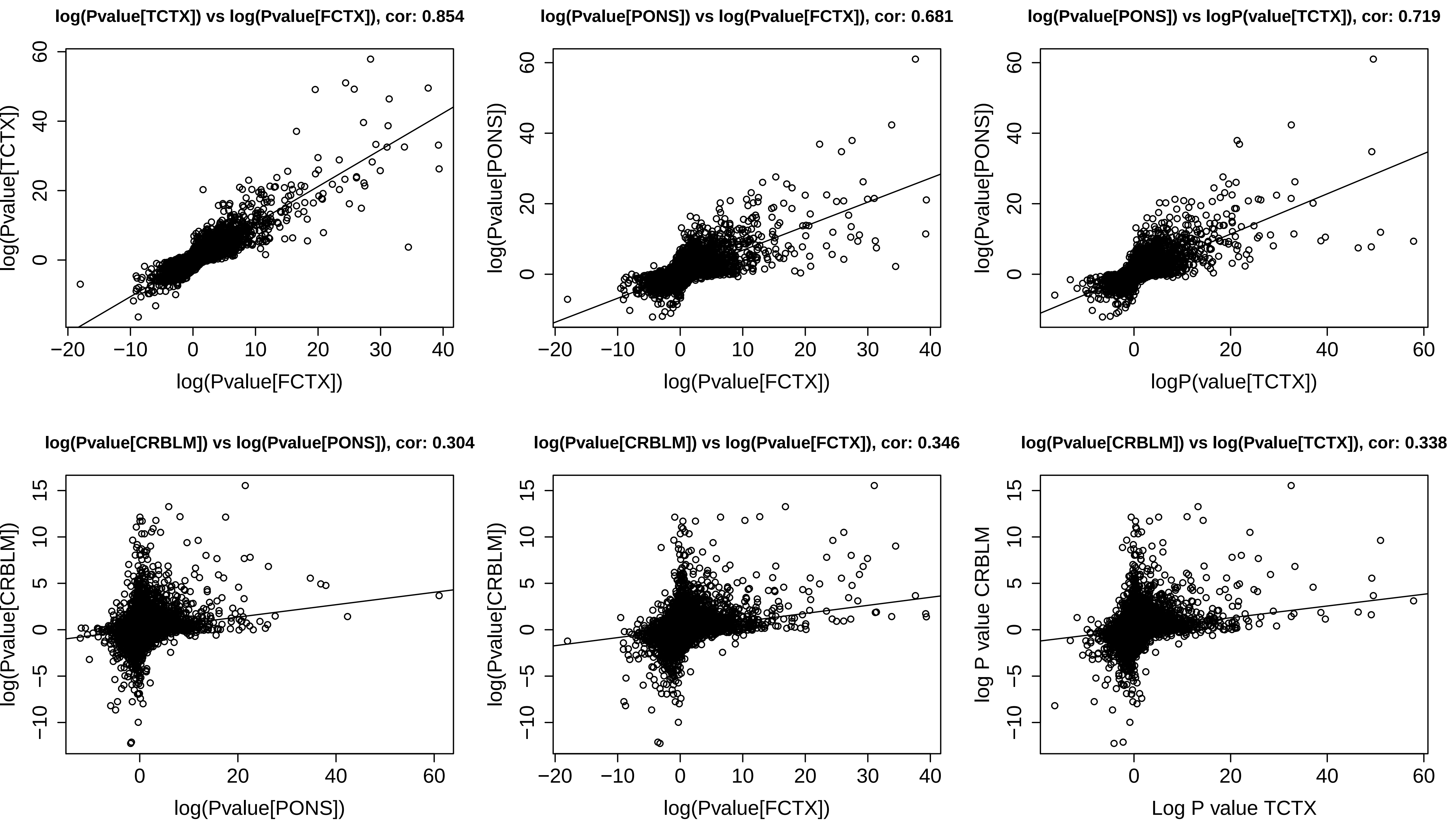
log(Pvalue[TCTX]) vs log(Pvalue[FCTX]), cor: 0.854
log(Pvalue[TCTX])
log(Pvalue[FCTX])
−20
−10
0
10
20
30
40
60
log(Pvalue[PONS]) vs log(Pvalue[FCTX]), cor: 0.681
log(Pvalue[PONS])
log(Pvalue[FCTX])
log(Pvalue[PONS]) vs logP(value[TCTX]), cor: 0.719
log(Pvalue[PONS])
logP(value[TCTX])
log(Pvalue[CRBLM]) vs log(Pvalue[PONS]), cor: 0.304
log(Pvalue[CRBLM])
log(Pvalue[PONS])
15
5
−5
−10
log(Pvalue[CRBLM]) vs log(Pvalue[FCTX]), cor: 0.346
log(Pvalue[CRBLM])
log(Pvalue[FCTX])
log(Pvalue[CRBLM]) vs log(Pvalue[TCTX]), cor: 0.338
log P value CRBLM
Log P value TCTX
