# Supplementary material for: Aging effects on DNA methylation modules in human brain and blood tissue
Source: Genome Biol. 2012 Oct 3;13(10):R97. doi: 10.1186/gb-2012-13-10-r97 (PMC4053733; doi:10.1186/gb-2012-13-10-r97)
Supplement: Additional file 3 — Module preservation analysis. The figures report the results of the module preservation analysis in the validation data sets. Each figure (page) corresponds to one validation data set. The left and right panels of each figure show the results for the Zdensity and medianRank statistics, respectively. The higher the value of the Zdensity statistic (and the lower the value of the median rank statistic), the stronger the evidence that the consensus module (based on the ten reference data sets) is preserved in the validation data set. The Zdensity statistic is based on a permutation test that allows one to establish significance thresholds (that are indicated by the horizontal lines at values 2 and 5 in the left panel). Values of Zdensity larger than 5 indicate moderate preservation while values below 2 indicate no evidence of preservation. [file gb-2012-13-10-r97-S3.PDF]

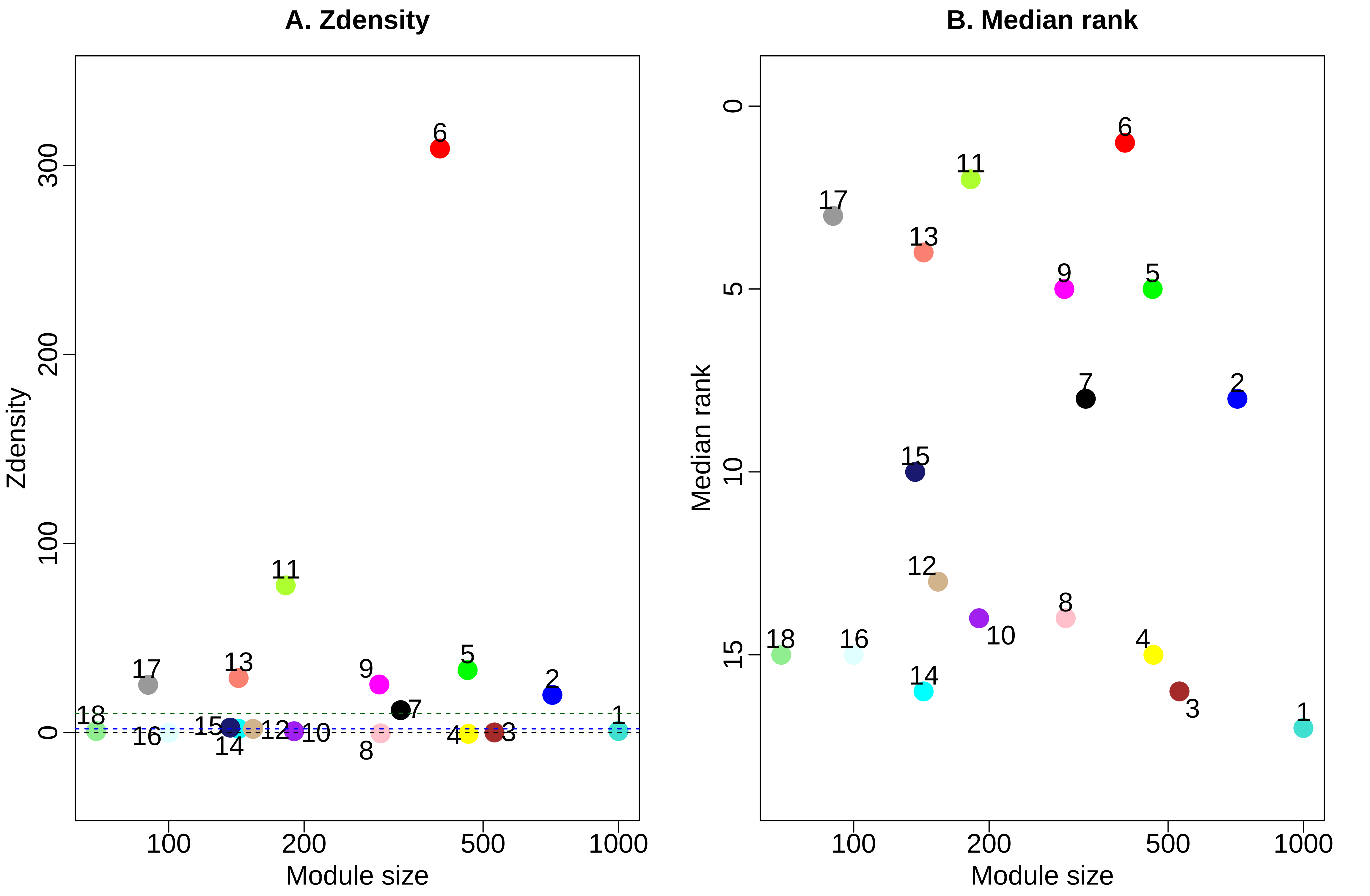

A. Zdensity
B. Median rank
Zdensity
Median rank
Module size
Module size
0
100
200
300
0
5
10
15
100
200
500
1000
100
200
500
1000
1
2
3
4
5
6
7
8
9
10
11
12
13
14
15
16
17
18

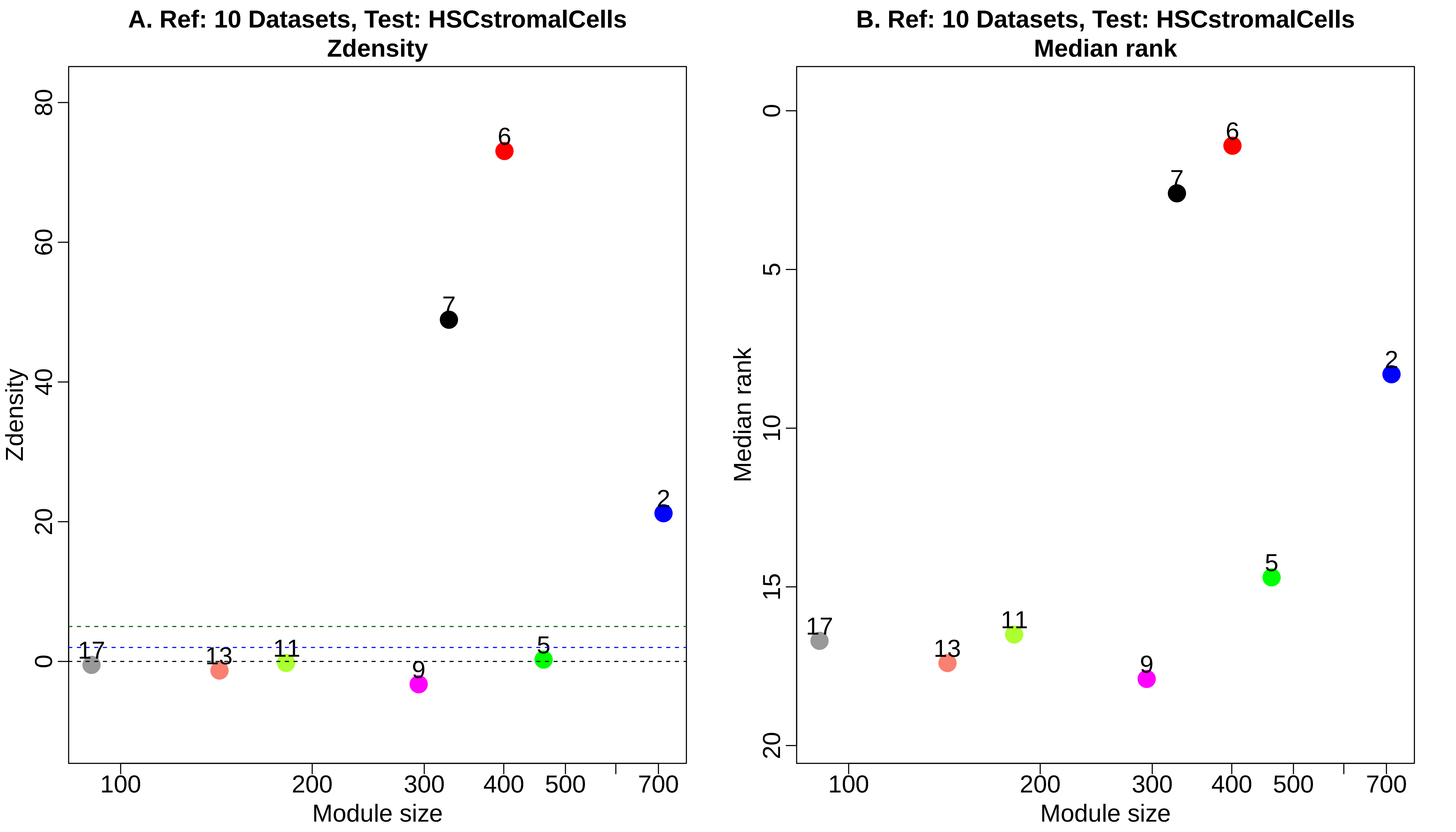
A. Ref: 10 Datasets, Test: HSCstromalCells
Zdensity
Zdensity
Module size
B. Ref: 10 Datasets, Test: HSCstromalCells
Median rank
Median rank
Module size
17
13
11
9
7
6
5
2
100
200
300
400
500
700
0
20
40
60
80
5
10
15

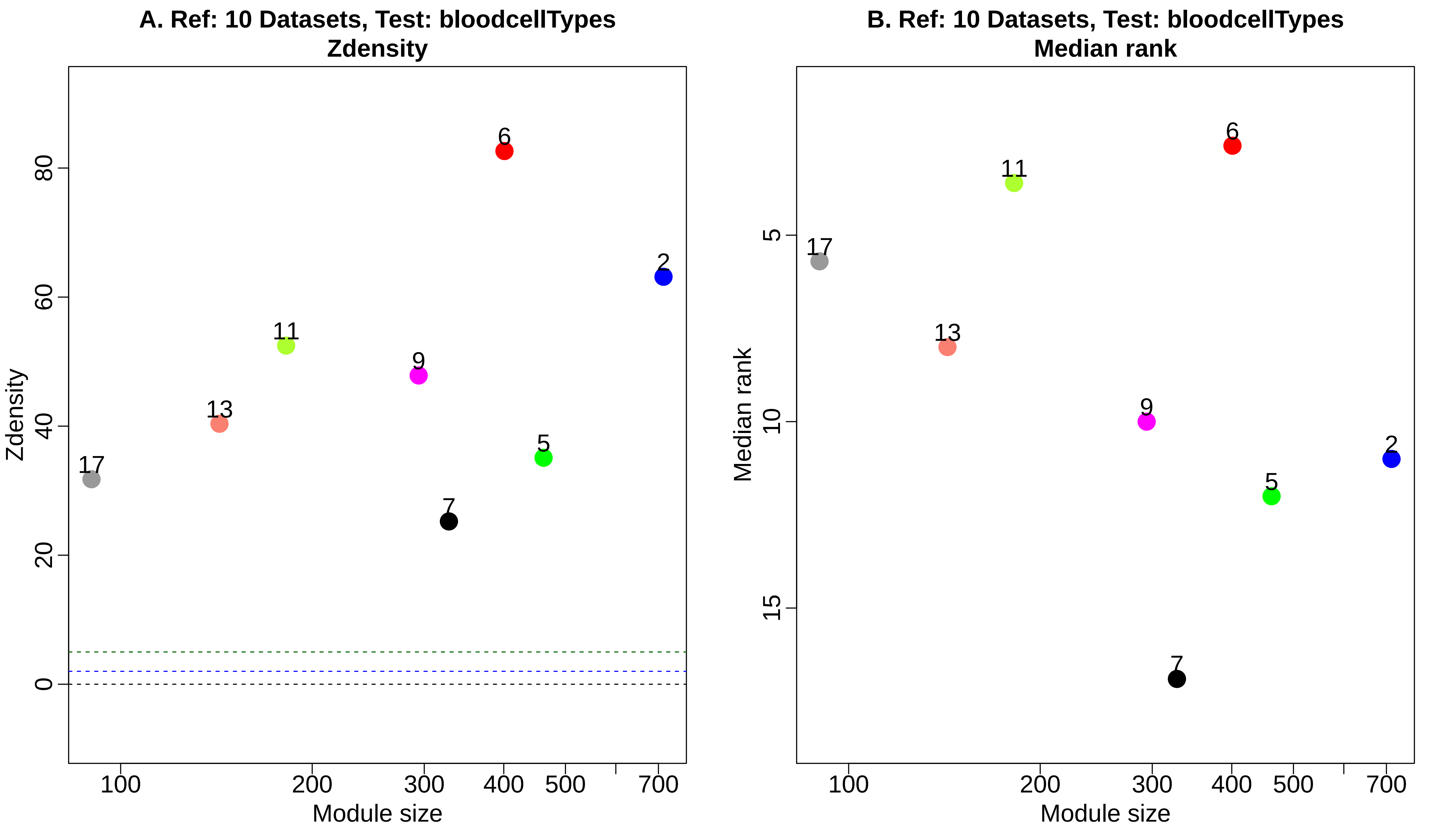

A. Ref: 10 Datasets, Test: bloodcellTypes
Zdensity
Zdensity
0
20
40
60
80
100
200
300
400
500
700
Module size
17
13
11
9
7
6
5
2
B. Ref: 10 Datasets, Test: bloodcellTypes
Median rank
Median rank
5
10
15
100
200
300
400
500
700
Module size
17
13
11
9
7
6
5
2

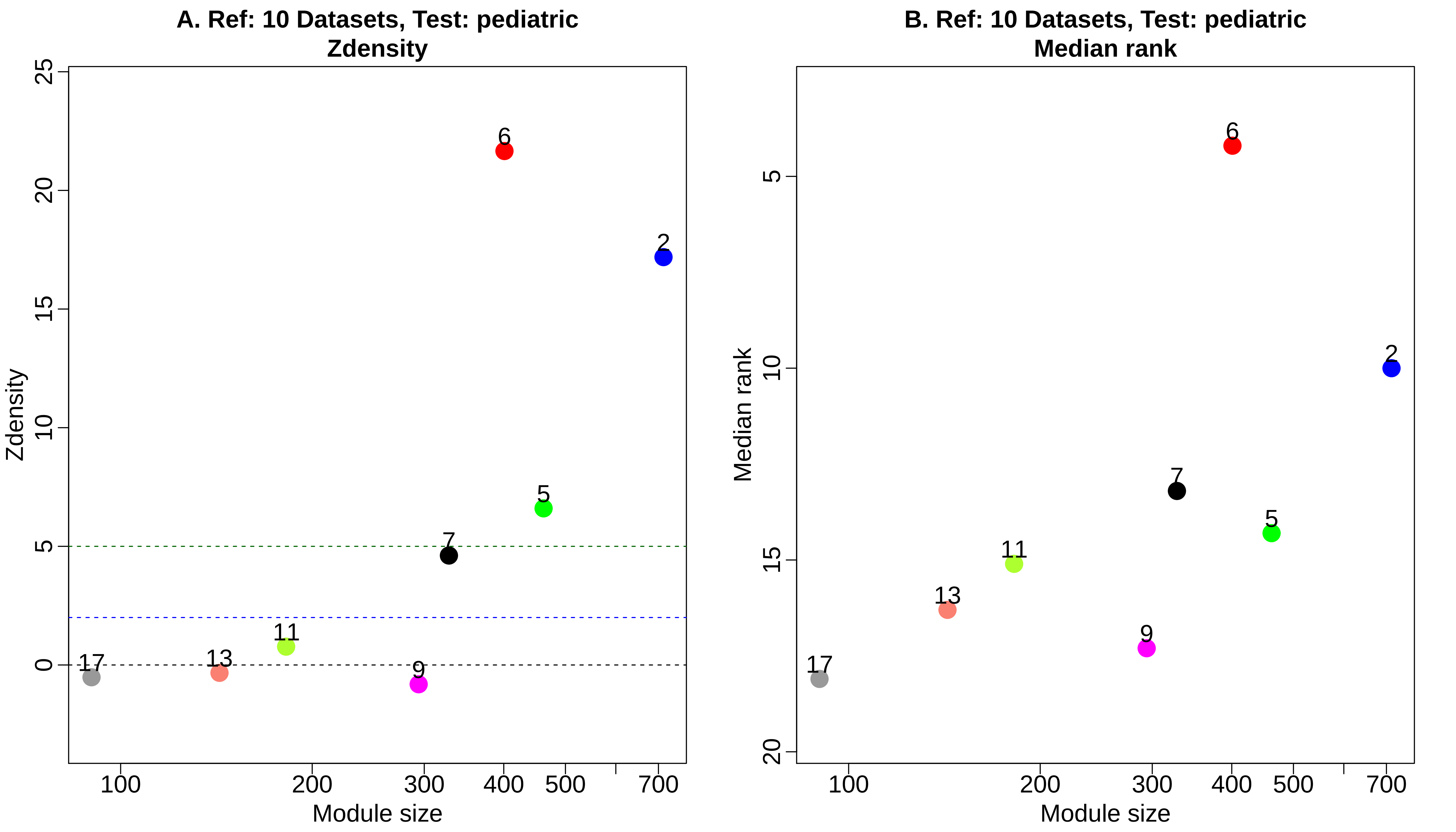
A. Ref: 10 Datasets, Test: pediatric
Zdensity
Zdensity
Module size
0
5
10
15
20
25
100
200
300
400
500
700
6
2
5
7
11
13
17
9
B. Ref: 10 Datasets, Test: pediatric
Median rank
Median rank
Module size
5
10
15
20
100
200
300
400
500
700
6
2
7
5
11
13
9
17

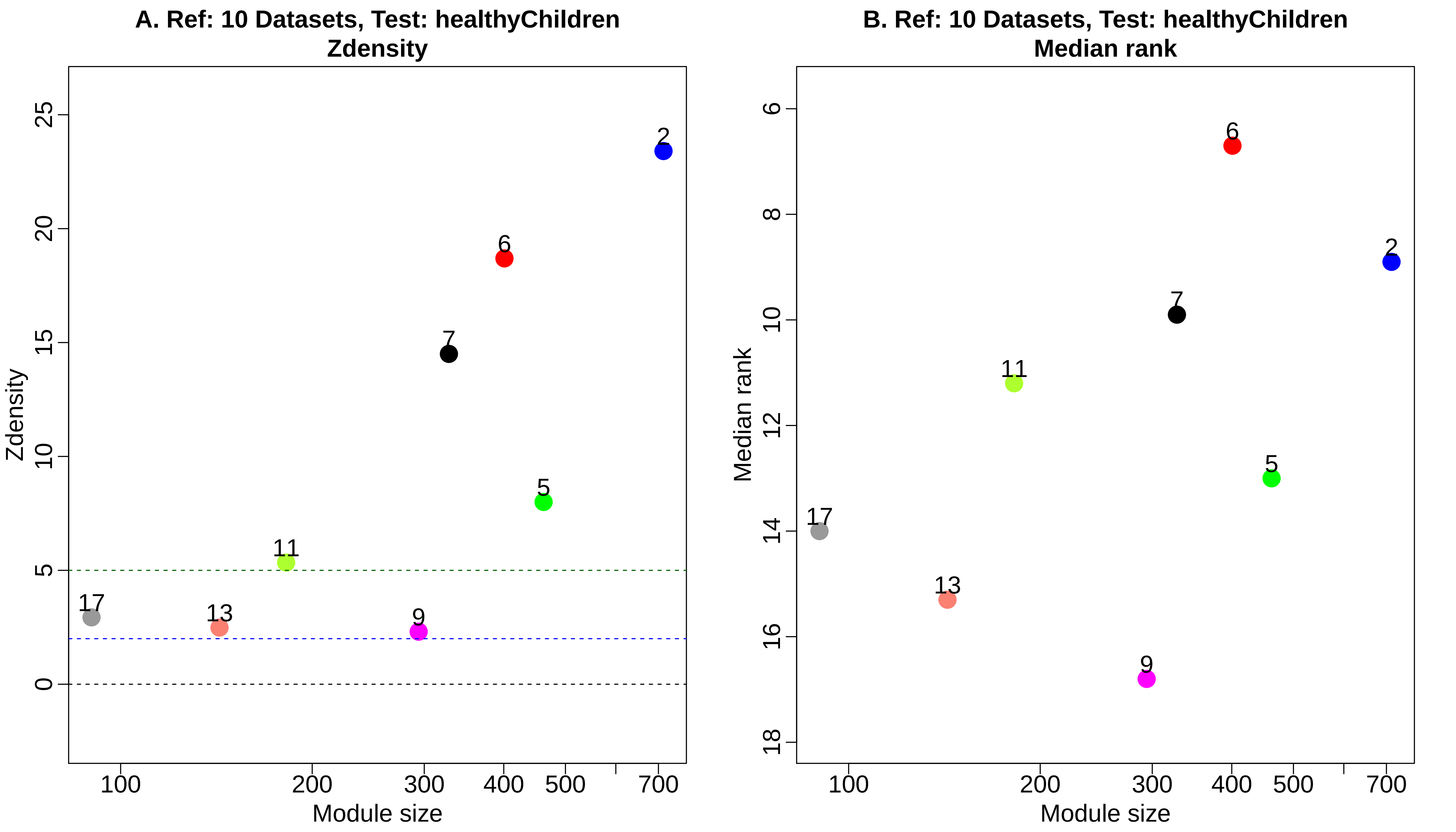
A. Ref: 10 Datasets, Test: healthyChildren
Zdensity
Zdensity
Module size
0
5
10
15
20
25
100
200
300
400
500
700
17
13
11
9
7
6
5
2
B. Ref: 10 Datasets, Test: healthyChildren
Median rank
Median rank
Module size
6
8
10
12
14
16
18
100
200
300
400
500
700
17
13
11
9
7
6
5
2

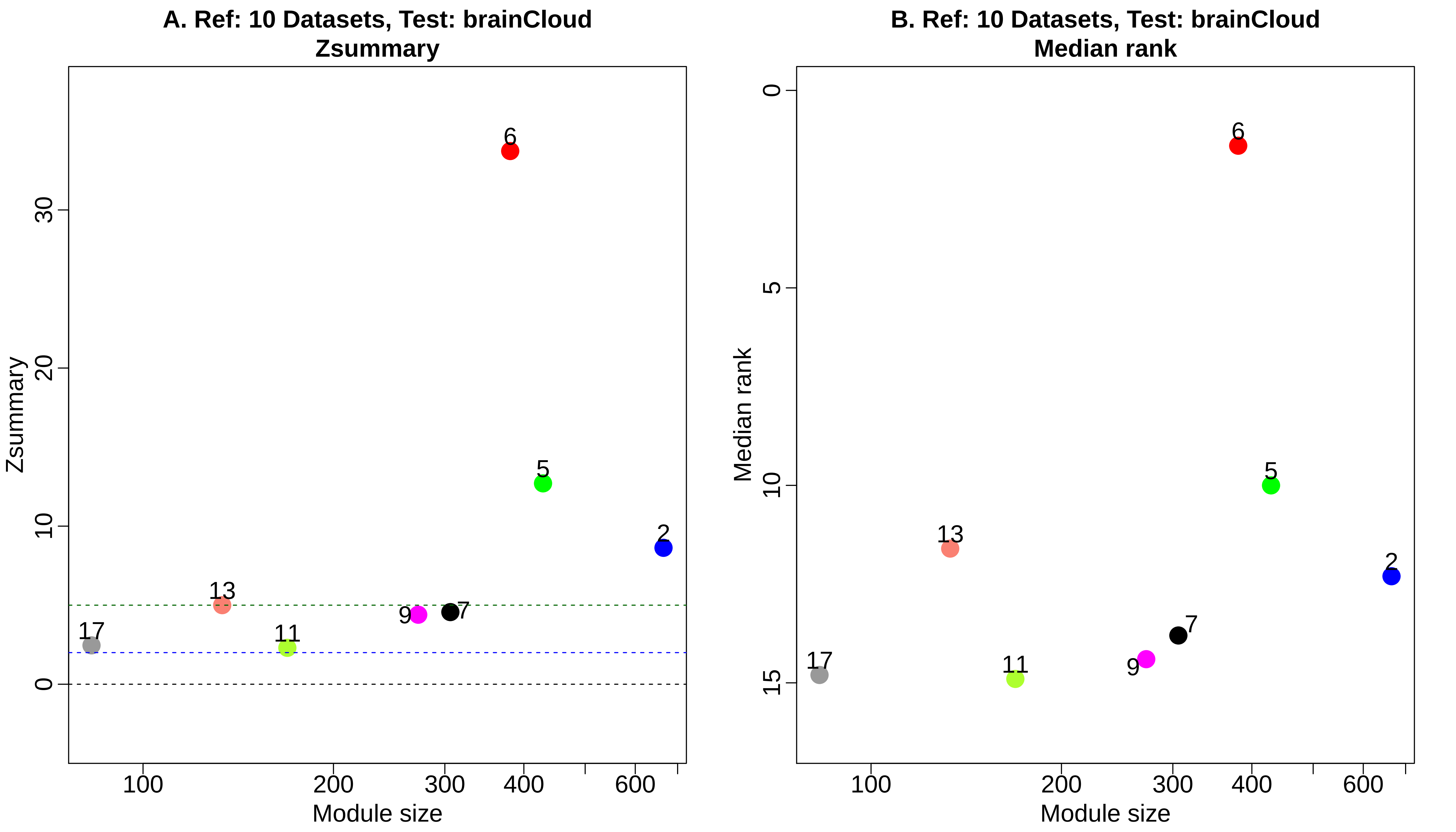

A. Ref: 10 Datasets, Test: brainCloud
Zsummary
Zsummary
0
10
20
30
100
200
300
400
600
Module size
17
13
11
9
7
6
5
2
B. Ref: 10 Datasets, Test: brainCloud
Median rank
Median rank
0
5
10
15
100
200
300
400
600
Module size
17
13
11
9
7
6
5
2
