# Supplementary material for: Aging effects on DNA methylation modules in human brain and blood tissue
Source: Genome Biol. 2012 Oct 3;13(10):R97. doi: 10.1186/gb-2012-13-10-r97 (PMC4053733; doi:10.1186/gb-2012-13-10-r97)
Supplement: Additional file 8 — Analysis overview. The figure shows the analysis steps of the consensus network analysis and their rationale. [file gb-2012-13-10-r97-S8.PDF]

Construct a co-methylation network for each of the 10 data sets and Corresponding robust similarity measures (Topological Overlap Matrices).
Rationale: make use of co-methylation patterns between CpGs

↓

Define a consensus network and consensus modules by aggregating the 10 TOM measures
Rationale: find shared consensus modules in blood and brain tissue

↓

Relate consensus modules to external information
Sample traits: age, gender
Gene Information: gene ontology (GO), cell markers (userListEnrichment)
Rationale: find aging related modules and understand their biological role

↓

Study the preservation of the aging module across different data
Rationale:
- in similar data: check robustness of module definition
- dissimilar data: Illumina 450K data, pediatric population, cell types, MSC

↓

Analysis of variance of module membership in the aging module
Sources of variation: Polycomb group target, CpG island, X-chromosome
Rationale: Understand relative contribution of factors
